# Self-assembled patient-derived tumor-like cell clusters for personalized drug testing in diverse sarcomas

**DOI:** 10.1016/j.xcrm.2025.101990

**Published:** 2025-03-06

**Authors:** Tian Gao, Xinyu He, Junyi Wang, Jiayong Liu, Xiongbing Hu, Chujie Bai, Shenyi Yin, Yunfei Shi, Yanmin Wang, Zhichao Tan, Fang Cao, Shu Li, Yan-Jie Shi, Ruifeng Xue, Juan Li, Yang He, Jiaxin Li, Huinan Lu, Hanshuo Zhang, Lu Zhang, Zhiwei Fang, Xinyu Wang, Mengmeng Liu, Wenjun Fu, Lei Tang, Buqing Ye, Zhengfu Fan, Jianzhong Jeff Xi

**Affiliations:** 1 State Key Laboratory of Natural and Biomimetic Drugs, Department of Biomedical Engineering, College of Future Technology, Peking University, Beijing 100871, China; 2 Key Laboratory of Carcinogenesis and Translational Research, Ministry of Education, Peking University Cancer Hospital & Institute, Beijing 100142, China; 3 Peking University Yangtze Center of Future Health Technology, Wuxi 214111, China; 4 GeneX Health Co., Ltd., Beijing 100195, China

**Keywords:** cancer, cancer models, high-throughput screening, sarcoma, PTC, personalized medicine, tumor microenvironment, off-label

## Abstract

Several patient-derived tumor models have emerged recently. However, soft tissue sarcomas (STSs) present a challenge in developing preclinical drug-testing models due to their non-epithelial and complex nature. Here, we report a model termed patient-derived tumor-like cell clusters (PTCs) derived from STS patients. PTCs result from the self-assembly and proliferation of mesenchymal stem cells (MSCs), epithelial cells, and immune cells, faithfully recapitulating the morphology and function of the original tumors. Through standardized culture and drug-response assessment protocols, PTCs facilitate personalized drug testing, evaluating hundreds of therapies within two weeks. Notably, PTCs exhibit 100% accuracy in distinguishing between complete or partial response and disease progression. We demonstrate the utility of PTCs in guiding chemotherapy selection for a patient with relapse and metastases following conventional therapy, who exhibited a positive response after non-conventional therapy identified through PTC. These findings underscore the potential of PTCs for prospective use in clinical decision-making regarding therapy selection.

## Highlights

• A sarcoma PTC model comprises primary tumor cells and endogenous stromal cells
• PTC enables the testing of hundreds of drugs in a standardized manner within 10 days
• PTCs demonstrate 100% accuracy in discriminating CR/PR from PD in clinical trials
• PTCs guide off-label use in a drug-resistant patient, who subsequently achieves PR

## Graphical abstract

**Figure:**
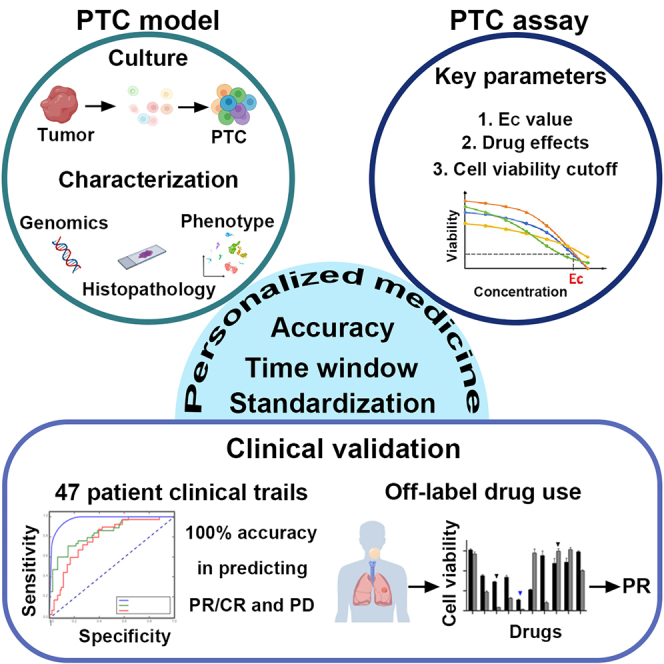

## Teaser

Personalized treatment for sarcoma is challenging due to its non-epithelial nature and biological complexity. Gao et al. show that a self-assembled system derived from patient-derived tumor-like cell clusters can serve as a valuable preclinical model for advancing personalized medicine in sarcoma.

## Introduction

Soft tissue sarcomas (STSs) are a heterogeneous group of malignant tumors originating from mesenchymal cells, representing approximately 1% of all malignancies.^1^^,^^2^ In children and adolescents, sarcomas account for about 20% of cancer-related deaths.^3^ With over 50 subtypes and high recurrence rates, STS presents a significant clinical challenge.^1^ While commonly used chemotherapeutics like doxorubicin and ifosfamide achieve a response rate of approximately 60% in early-stage diagnosis, this drops dramatically to 10% in advanced stages.^4^^,^^5^^,^^6^^,^^7^ The substantial molecular and clinical heterogeneity of STS complicates mechanism investigation and underscores the importance of personalized chemotherapy selection.

Recent advancements in 3D culture technologies have led to the development of sarcoma spheres or organoids.^8^ However, existing methods, including pellet culture, spinner culture, hanging drop, and others, have many limitations.^9^^,^^10^^,^^11^^,^^12^ For instance, spheres derived from cell lines often fail to recapitulate the characteristics of original tumors, while those from fresh tumor samples show low culture success rates (∼30%–79%) and proliferation rates, limiting their clinical applicability for drug screening.^13^^,^^14^ The use of Matrigel or hydrogel as an extracellular matrix substitute also hinders the growth of immune and other microenvironment cells.

Patient-derived organoids (PDOs) derived from tissue-derived adult stem cells have been instrumental in modeling treatment responses in various cancers.^15^^,^^16^^,^^17^^,^^18^ Up to now, about 20 studies to assess the clinical validity of PDOs as a predictive biomarker for treatment response in the clinic have been reported.^19^ However, PDOs had many limitations in clinic applications. First, the accuracy of PDOs in predicting clinical responses to chemotherapies remains controversial^20^^,^^21^; colorectal cancer PDOs, one of most successful PDOs, can predict the drug response of irinotecan with the high accuracy but failed to predict the outcome of patients treated with first-line 5-fluorouracil and oxaliplatin.^20^ Second, the definition of organoid is unclear but with the characteristics of matrix used as an essential component. The culture medium may vary, and, even within the same tumor type, medium compositions and culturing techniques differ.^19^ Thus, implementing sarcoma PDOs in personalized medicine faces the following challenges, including the standardization of cell culture conditions and drug concentrations, balancing culture time window and throughput in drug testing, and accurately predicting clinical outcomes.^19^^,^^20^^,^^22^^,^^23^

**Figure 1:**
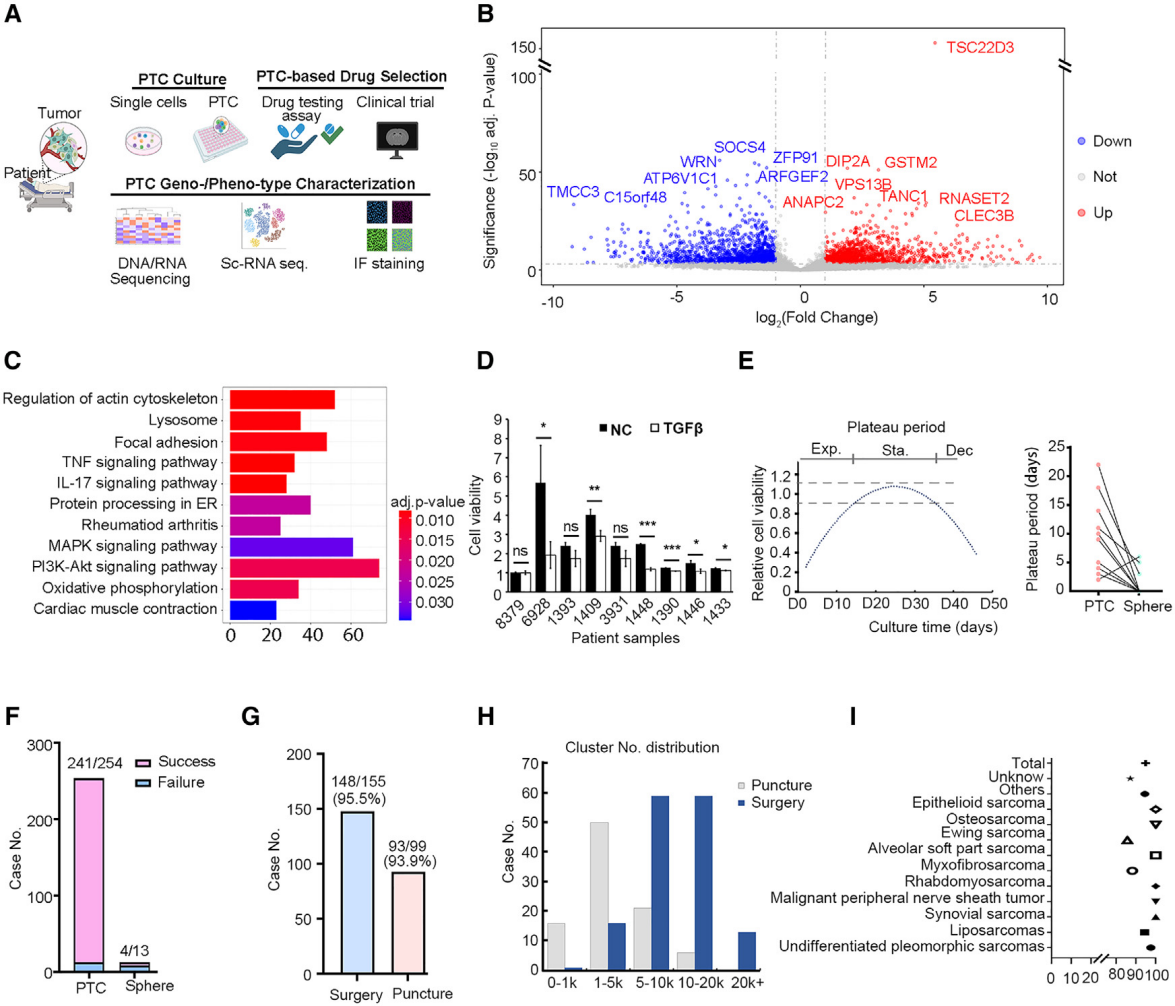
Establishment of PTC *in vitro* tumor models (A) Overview of the process for generating PTCs and conducting personalized drug testing. (B) Volcano plot showing differential gene expression in tumor samples compared to spheres (*n* = 2,996, adjusted *p* value < 0.001). (C) KEGG overrepresentation analysis of differentially expressed genes, adj. *p* value means adjusted *p* value. (D) Cell viability between sarcoma PTCs with or without TGF-β. Each group has three replicates (*n* = 3, data are represented as means ± STD). Paired sample t test is used to compare the difference between negative control group and TGF-β group. ns means *p* value > 0.05, ∗ means *p* value ≤ 0.05, ∗∗ means *p* value ≤ 0.01, and ∗∗∗ means *p* value ≤ 0.001.STD, standard deviation. (E) The schematic exponential, stationary, and decline phase (left) and the plateau period comparison of the sphere and PTC model (right). (F) The numbers of culturing and success rate of the sphere and PTC model. (G) The success rate and source of PTC culturing. (H) The distribution of numbers from different origin PTC clusters. (I) The success rate of PTC culturing among different subtypes.

To address these challenges, we have developed an STS patient-derived tumor-like cell cluster (PTC) model by adapting and optimizing culture methods.^23^^,^^24^ STS PTCs not only establish a broad sarcoma classification with a 95% success rate but also accurately reflect the molecular and cellular components of original sarcoma tissues (Figure 1A). In a non-intervention clinical cohort study involving 47 patients, our method demonstrated 100% accuracy in discriminating between complete/partial response (CR/PR) and progressive disease (PD). Furthermore, we achieved significant lesion reduction in a drug-resistant synovial sarcoma (SS) patient, resulting in a PR, using a non-conventional therapy guided by PTC results.

## Results

### Establishment of STS PTC models

To achieve long-term maintenance and expansion of primary tumor cells and endogenous immune cells, we optimized a Matrigel-free and serum-free culture media for STS. First, we performed RNA sequencing (RNA-seq) on original tumor samples and cultured spheres derived from three patients’ sarcoma samples to identify key pathways or genes involved in tumor development. Analysis revealed a significant number of differentially expressed genes (DEGs) in the sphere models compared to the original tumor tissue (adjusted *p* value < 0.001). Enrichment analysis of the Kyoto Encyclopedia of Genes and Genomes (KEGG) pathway for 2,996 DEGs showed significant differences in pathways such as tumor necrosis factor, mitogen-activated protein kinase, and PI3K-Akt between the sphere models and original tumors (Figures 1B and 1C). Several top-listed DEGs were highly related to transforming growth factor β (TGF-β) signaling (Figure 1B), including *TSC22D* and *CLEC3B*, known to regulate cell differentiation in response to TGF-β and inhibit myogenic differentiation.^25^

Secondly, we integrated DEGs, existing literature, and the previously described PTC culture medium^23^^,^^24^ to develop an optimized sarcoma PTC medium (Table S1). This medium eliminated matrix, collagen, or serum, which contains undefined extrinsic factors, and included essential factors such as epidermal growth factor (EGF), fibroblast growth factor (FGF), and Y-27632 to promote PTC formation. Notably, we removed TGF-β from the culture medium, since it significantly inhibited sarcoma PTC growth (Figure 1D). In addition, a number of compounds essential for the generation of organoids, such as A83-01, SB202190, Noggin, Wnt3a, and R-spondin, were removed from the sarcoma PTC medium, since they had negligible effects on sarcoma PTC growth, whereas materials with hydrophobicity were implemented to modify the culture plate or chip in order to enhance the formation of sarcoma clusters (Figure S1A).

Using these optimized approaches, we dynamically studied PTC generation through a high-content imaging system. PTC formation primarily occurred through the self-assembly of dissociated primary cells into clusters, completing the process within the initial 2 days (Figure S1B and Video S1). PTC growth exhibited three stages: exponential, stationary, and decline, with cell viability 7-fold that of conventional spheres (Figures 1E and S1C). Notably, PTCs displayed a more extended growing stage and plateau period compared to spheres. Conventional spheres typically began to shrink between the 3^rd^ and 7^th^ days with a 1- to 2-day plateau period. In contrast, PTCs exhibited exponential growth in the first week followed by a plateau period lasting from 5 to 25 days (Figure 1E). The success rate of generating PTCs was 94.9% (241/254), higher than that of spheres (30.8%, 4/13) (Figure 1F).

Video S1. Process of self-assembly from dissociated primary cells into PTC, related to Figure 1

We then conducted comparative analyses between PTCs and tumor spheres, as well as between PTCs and paired tumor samples. A total of 3,740 DEGs were identified between PTCs and tumor spheres, while 1,222 DEGs were identified between PTCs and tumor samples (Figures S1D and S1E). Notably, genes that were significantly downregulated in PTCs compared to tumor samples were predominantly endothelial related, including *VWF* and *CD34*. This finding aligns with the observed absence of blood vessels in PTCs (Figure S1E).

From 2019 to the present, we obtained 254 samples (155 surgical, 98 puncture, and 1 ascites sample) to generate PTCs, covering tens of sarcoma classifications, with an overall success ratio of 94.9%, ranging from 85.7% to 100% (Figures 1F–1I and Table S1). The number of clusters generated varied from 500 to 50,000 in a week, depending on the sampling resource (Figure 1H). Surgical samples predominantly had 5,000–20,000 clusters, while biopsy samples exhibited fewer clusters. These unique properties, including Matrigel-free culture, long plateau period, high success rate, and increased cluster numbers, make the PTC model highly suitable for personalized drug testing within a 2-week time window.

### Genomic consistency of PTCs with original tumors

**Figure 2:**
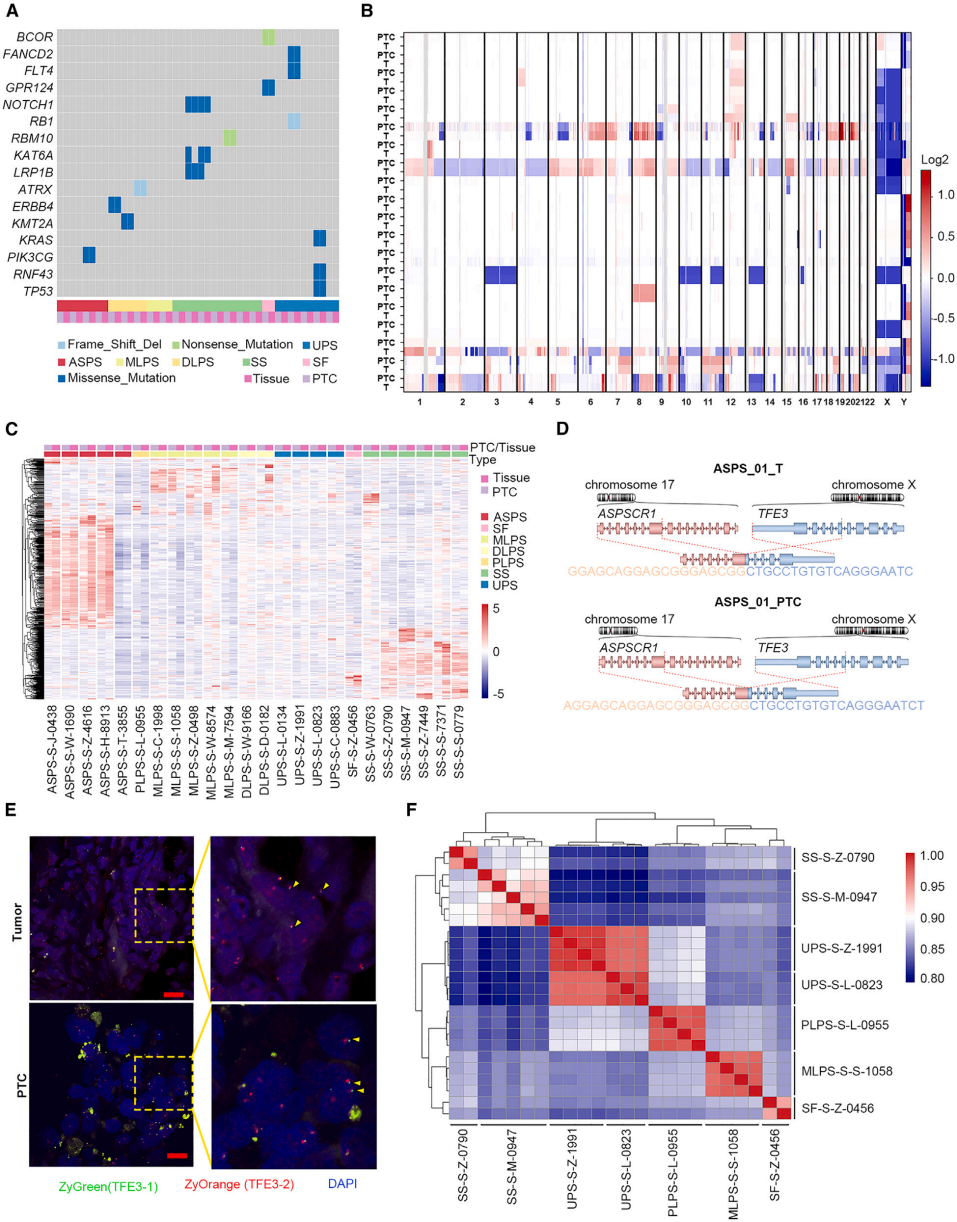
Genomic comparison between tumor biopsies and corresponding PTCs (A) Overview of somatic mutations identified in 22 patients with six different soft tissue sarcoma types. (B) Copy-number variation (CNV) comparison between PTCs and corresponding tumors. Columns represent genomic positions from chromosomes 1 to 22. (C) Consistency of transcriptional expression between primary tumor and PTC. (D) Detection of *ASPSCR1-TFE3* fusion in patient with alveolar soft tissue sarcoma. (E) Validation of the fusion using a TFE3 probe by FISH staining in alveolar soft tissue sarcoma. Scale bars, 20 μm. (F) Gene expression correlation in PTCs between different wells.

We conducted a comprehensive analysis of the genomic consistency between PTCs and their parental tumor samples. Somatic mutations and DNA copy-number variations (CNVs) of PTCs were assessed compared to the original tumors. Genomic DNA from 22 pairs of PTCs and tumor samples from six different types of STS, along with their matched normal samples, was isolated for targeted DNA sequencing (DNA-seq) analysis. The mutation pattern of PTCs was consistent with that of the original tumor in all 22 patients (Figure 2A). Notably, nine patients showed no tumor-specific gene mutations above the threshold (Figure 2A), consistent with the low mutation load in sarcomas.^26^^,^^27^ Low-pass whole-genome sequencing (WGS) analysis revealed consistent CNV characteristics in PTCs and original tumors (Figures 2B and S2A). For instance, dedifferentiated liposarcomas (DLPSs) exhibited amplification of the long arm of chromosome 12 and *CDK4/MDM2*, which was also observed in corresponding PTCs (Figure S2A).

Next, we compared the gene expression profiles of 24 pairs of PTCs and their parental tumors from seven types of STSs using RNA-seq (Figure 2C). Different types demonstrated highly specific but consistent transcriptional expression patterns between PTCs and their corresponding tumor samples (Figure 2C). For instance, *MDM2* and *CDK4* were highly expressed in DLPS in both PTCs and tumors (Figure S2B), consistent with clinical diagnostic criteria.^28^^,^^29^^,^^30^ Additionally, both tumors and PTCs of alveolar soft tissue sarcoma (ASPS) highly expressed *PSAP*, *MDK*, and *HIF1A* (Figure S2C), known to be related to the differentiation of mesenchymal stem cells.^31^

Furthermore, STSs are characterized by specific gene fusions.^4^^,^^32^ We examined fusion transcriptions in 23 pairs of PTCs and the original tumors (Table S2), revealing 100% consistency of both positive and negative gene fusions. For example, *SS18-SSX1/2/2B* or *NAB2-STAT6* fusion existed in SS, and *ASPSCR1-TFE3* fusion in ASPS, whereas no/low fusion was detected in DLPS, pleomorphic liposarcomas, and undifferentiated pleomorphic sarcomas (UPSs) (Figure 2D and Table S2). Fluorescence *in situ* hybridization (FISH) validated *ASPSCR1-TFE3* fusion transcripts in both tissue samples and PTCs derived from patient ASPS-S01 (Figure 2E). Together, these data demonstrated the ability of PTCs to accurately recapitulate the key gene-fusion features of the original tumors.

To ensure the consistency of PTCs across different wells for subsequent drug screening, we examined the consistency in somatic mutations and transcriptome profiles between PTCs in individual wells. Pairwise overlap analysis demonstrated that the proportions of common somatic mutations between different wells consistently exceeded 100% for the top 15 genes (Figure S2D). Additionally, gene expressions exhibited high correlation between different wells (Figure 2F). These findings indicate that PTCs from various wells maintain relatively consistent biological characteristics during the culture process, making them well-suited for high-throughput drug testing.

### Consistency of pathological features, cell composition, and immune infiltration in PTCs compared with original tumors

**Figure 3:**
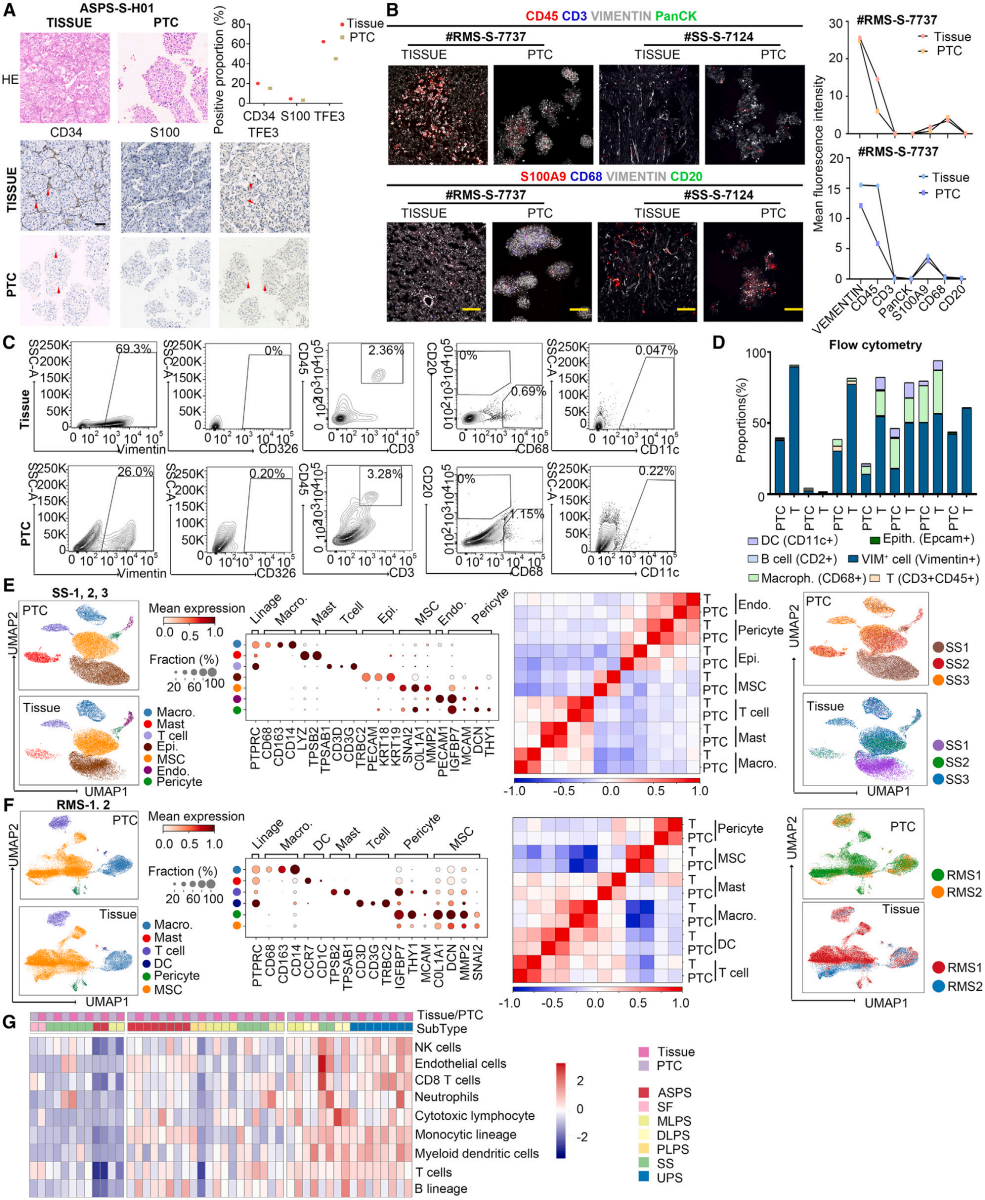
Cell composition and immune infiltration comparison between tumor and corresponding PTCs (A) H&E and IHC staining of tumors and corresponding PTCs from patients with ASPS and positive proportion of CD34, S100, and TFE3. Scale bars, 100 μm. (B) Immunofluorescence staining of tumors and corresponding PTCs showing immunological cells and tumor cells and mean fluorescence intensity of each marker. Scale bars, 100 μm. (C and D) Comparison of different cell proportions between PTCs and tumors using flow cytometry. Samples from the 7^th^ day of incubation were used for experiments. (E and F) Uniform manifold approximation and projection (UMAP) plots of major cell types identified in three pairs of SS samples and two pairs of RMS samples and dot plots of marker genes of each cell type. Dot plots show marker gene expression levels normalized by *Z* score (dot color), and dot size represents the fraction of expression in one cell type. High correlation between paired tumor samples and PTCs is observed when scRNA-seq data of both pairs of PTCs and parental tumor samples are merged and analyzed. No batch effect is observed. (G) MCP-counter deconvolution result of the transcriptomes of STS tumor and PTCs, roughly categorized into three types based on immune infiltration patterns.

Pathological examination is crucial for the differential diagnosis of STS.^33^ Both hematoxylin and eosin (H&E) and immunohistochemistry (IHC) staining results showed consistency between original tumors and PTCs derived from a group of patients with six different sarcoma types (Figures 3A and S3A). For instance, an ASPS patient (ASPS-S-H01) exhibited a well-defined nest pattern of cells with abundant pink cytoplasm and a pseudoalveolar appearance in both tumor and PTC samples (Figure 3A). The TFE3 marker of ASPS was diffusely positive in nuclear localization in both tumor and PTC samples (Figure 3A), indicating retention of this important molecular feature in the PTC model.^34^ Pathological consistency between PTCs and original tumor samples was also observed in other sarcoma classifications, such as malignant solitary fibrous tumor (Figure S3A).

Immunofluorescence staining revealed that PTCs contained CD45^+^ immune cells and/or Vimentin^+^ fibroblast cells (Figures 3B and S3B). All ten cases demonstrated that PTCs highly maintained the cell components present in their original tumors (Figures 3B and S3B). For example, rhabdomyosarcoma (RMS) RMS-S-7737 was rich in immune cells and low in fibroblast cells, whereas SS-S-7124 included low immune cells and high fibroblast cells. Furthermore, flow cytometry assays quantitatively analyzed the proportions of immune cells in PTCs and the original tumors from seven patients (Figure 3C and Table S3). The immune components in PTCs were roughly in the same proportion as the original tumor samples, suggesting that PTC accurately recapitulated the patient’s immune infiltration microenvironment (Figures 3C and 3D and Table S3). Thus, these results underscore the ability of the PTC model to faithfully recapitulate the sarcoma microenvironment in tissue samples.

Next, we conducted single-cell RNA sequencing (scRNA-seq) analysis of SS and RMS samples and examined the consistency of PTCs and the original tumors at a single-cell resolution (Figures 3E, 3F, S3D, S3E, and S3F). After quality control and filtration, we obtained 41,129 and 55,363 high-quality cells in each subtype, respectively, and categorized them into different cell types through unsupervised graph-based clustering. We identified diverse cell types of endothelial cells, pericyte cells, epithelial cells, T cells, Tregs (regulatory T cells), macrophages, dendritic cells, mast cells, plasma cells and tumor cells (MSC) (Figures S3E and S3F), similar with other sarcoma datasets.^35^^,^^36^^,^^37^ The cellular patterns of PTCs and parental tumors exhibited strongly consistent interpatient heterogeneity (Figures 3E and 3F). Furthermore, merging data analysis demonstrated high Spearman correlation coefficients between paired PTCs and patient samples for each cell type, falling within the range of 0.9–0.95 (Figures 3E and 3F). Interestingly, we observed three states, including progenitor, proliferating, and differentiated clusters (Figure S3G), in RMS tumor cells with each corresponding to the higher score, when we scored the data with characterized gene sets.^38^

For a deeper understanding of the tumor microenvironment in STS samples and PTCs, we applied the microenvironment cell populations-counter (MCP-counter) method^39^ to the transcriptomes of PTCs and original tumors. Immune infiltration patterns were consistent among these 24 pairs of samples, classifying into three groups: high, intermediate, or low immune infiltration (Figure 3G), which aligned well with previous classifications.^40^ Also, we used another tool, xCell to evaluate a broader range of cell compositions.^41^ The immune scores and the enrichment of multiple immune cell components indicated that these samples could be broadly categorized into three groups consistently (Figure S3H).

### Establishment of PTC as a drug-testing platform

**Figure 4:**
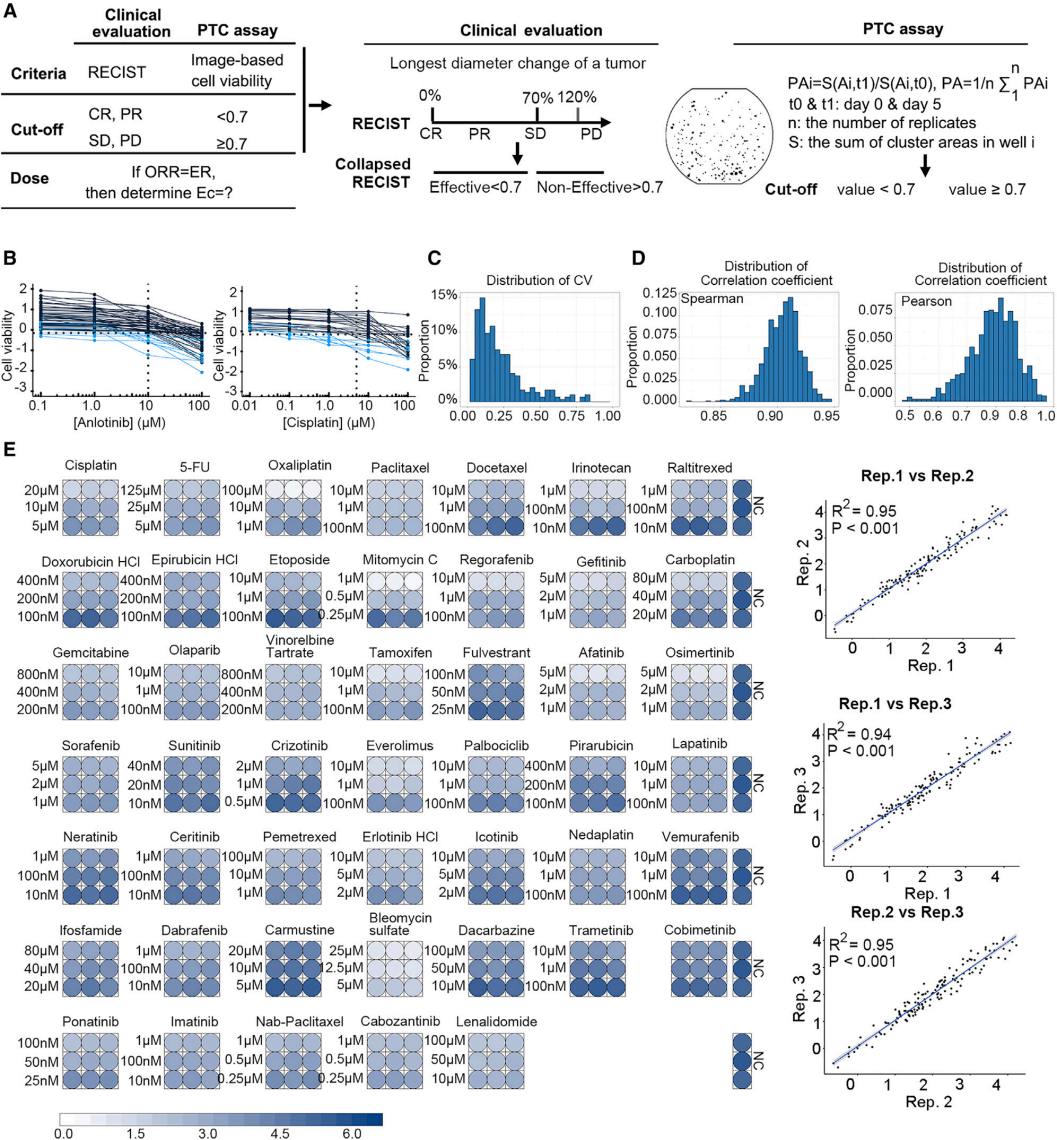
The establishment of the PTC system for personalized drug testing (A) Three key parameters in the PTC drug-testing assays (top) and a representative image of PTC clusters and the formula to calculate their cell viability (down). (B) Examples of determining the Ec of chemotherapies. The dotted line represents the cutoff value of 0.7. The blue line indicates effective tests below 0.7. (C) Distribution of CVs in the drug-sample pairs in the STS group. *n* = 337 drug-sample pairs. (D) Distribution of Spearman and Pearson correlation coefficients in a 1,000-fold random sampling strategy for two results in three parallel drug sensitivity tests. *n* = 337. (E) PTC drug-testing assays in a multi-well chip.

Prior to validating the accuracy of PTCs’ clinical predictions, we then develop a personalized drug-testing assay referring to the real-world clinical evaluation scenarios. According to response evaluation criteria in solid tumors (RECIST), we defined three crucial parameters for the PTC drug-testing assay: drug effects, cell viability cutoff, and drug efficacy concentration (Ec value) (Figure 4A), as described previously.^23^ In essence, we quantified the drug effect by measuring the area of all PTC clusters within a well (Figures 4A and S4A). Photographs were taken at day 0 and day 7 for all clusters in each well, and only clusters with diameters exceeding 40 μm were selected for calculating cluster areas. The cell viability after applying a specific drug (drug A) was calculated using the following formula:

$$p_{Ai}=S_{Ai,t1}/S_{Ai,t0},p_{A}=\frac{1}{n}\sum_{i=1}^{n}p_{Ai}\text{,}$$

where S_A_ represents the sum of cluster areas in well i, n is the number of replicates, and **t**_**0**_ and **t**_**1**_ are the time points when the areas were measured. We utilized the same cell viability value of the negative control as a quality control: if the cell viability score $p_{NC}$ was less than 0.9, indicating that the PTC was possibly in the decline phase, the PTC test was discarded. The cell viability cutoff value was then defined on the basis of the objective overall response rate (ORR). Categorizing cell viability results into two groups: effective drug if $p_{A}<0.7$ and not effective drug if $p_{A}\geq 0.7$ (Figures 4A and S4B), regardless of the combined or single therapies (Figure S4B).

Furthermore, we determined the PTC Ec value for drug A based on its clinical efficacy. Ten chemotherapeutic drugs were tested in the study, with 3–6 concentration gradients tested for each drug. The cell viability of PTCs was observed at different concentrations for each drug. By fixing the cutoff at 0.7, we determined the Ec of a drug as the concentration at which the effective rate in the PTC assay was closest to the ORR reported in relevant clinical trials (Figure 4B). The Ec values for all drugs used in this study were determined (Figure S4C) and summarized in Table S4.

### Preclinical evaluation of PTC as a tool for personalized drug testing

To assess the cell viability consistency across the wells prior to clinical evaluation, we initiated drug testing using PTC samples from 134 patients, encompassing 15–22 drugs per patient with three replications for each drug-sample pair. This yielded a total of 337 drug profiles. The mean coefficient of variation (CV) of drug sensitivity between wells was 0.23, with 91% of the CV being less than 0.50, underscoring the biological repeatability and consistency of drug sensitivity between wells (Figure 4C). Additionally, correlation coefficients were calculated for two randomly selected results from the 337 experiments and repeated 1,000 times. The Pearson correlation coefficient for multiple samplings was 0.80 with a standard deviation of 0.08, while the Spearman correlation coefficient had a mean value of 0.90 with a standard deviation of 0.02. These results indicate that biological replications for the same drug-sample pair were highly consistent (Figure 4D).

Subsequently, we demonstrated the feasibility of employing PTCs for high-throughput drug screening. In a multi-well chip, 423 PTC assays were conducted, with an average of 50–100 PTCs cultured in each well at three drug concentrations ranging from 0.01 to 125 μM, starting with a 0.25 g myxoid liposarcoma sample from patient GS-X-0805 (Figure 4E). The quality of the assay was confirmed by the correlation of cell viability between technical replicates (Pearson correlation = 0.95, 0.94, and 0.95, *p* values < 0.001), demonstrating high reproducibility (Figure 4E). Thus, the PTC assay can be applied to assess the activity of over hundreds of chemotherapy drugs as a preclinical model.

### PTCs accurately and quantitatively recapitulate the clinical outcomes of STS patients

**Figure 5:**
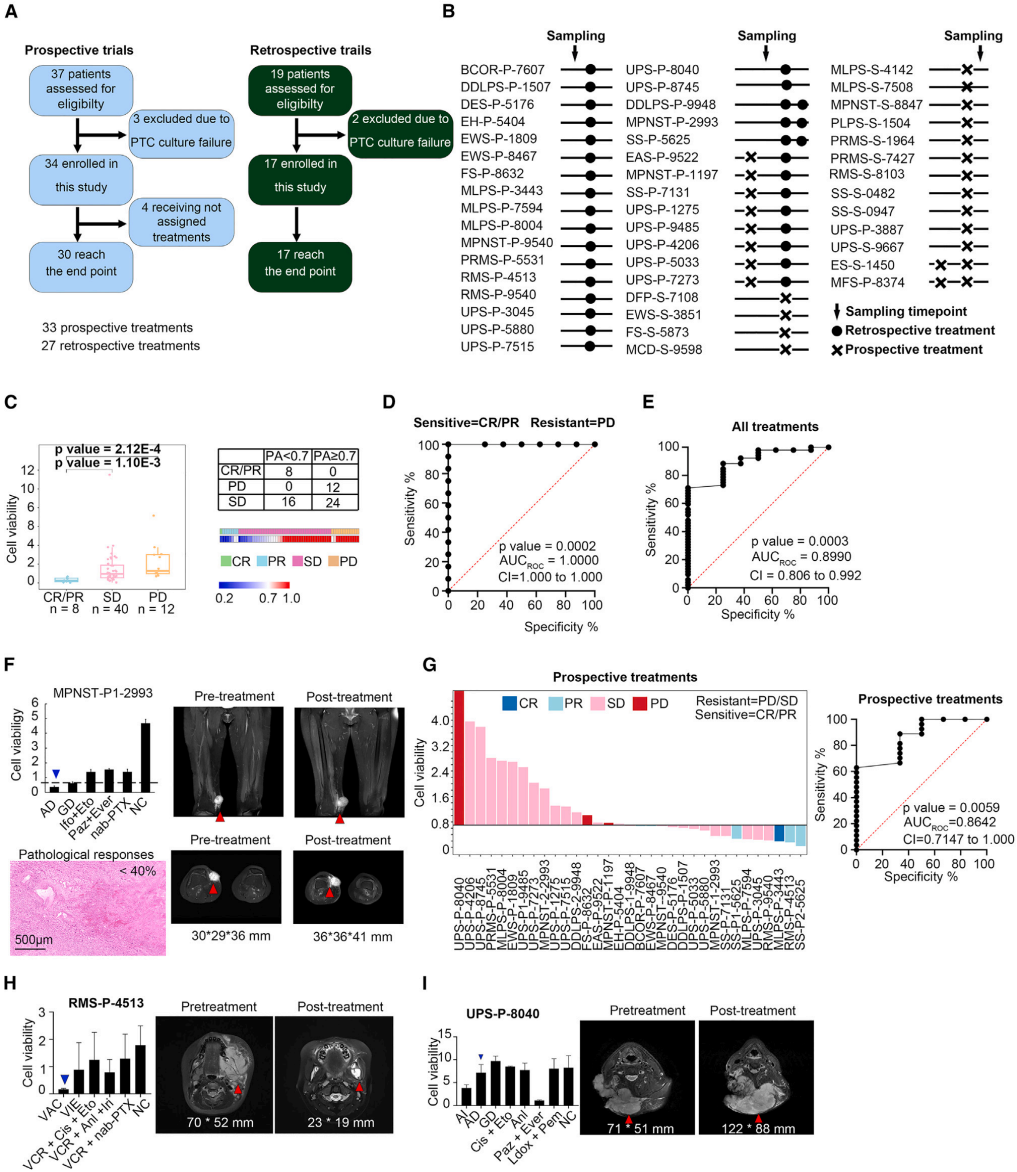
PTCs reflect the clinical outcomes of representative patients with STS (A) Flowchart illustrating the validation procedure. (B) Timeline illustrating 47 enrolled patients receiving retrospective and/or prospective treatments in relation to the designated sampling point. (C) Boxplot of the drug test results for 60 samples of CR/PR (*n* = 8), SD (*n* = 40), and PD (*n* = 12) on the basis of PTC assays. Groups were compared using two-sample Wilcoxon’s test (called as Mann-Whitney test). A 2 × 3 contingency table and a heatmap summarize *in vitro* drug responses based on PTC testing and RECIST clinical outcome. (D) Data presented as an ROC curve of 20 prospective treatments in the CR/PR and PD groups. (E) Data presented as an ROC curve of all 60 treatments in the CR/PR and SD/PD groups. (F) The drug-response profile of PTCs from patient MPNST-P1-2993. PTC cell viability value was 0.36 after AD, showing drug-sensitive but clinically SD by CT images (*n* = 3, data are represented as means ± STD). H&E staining of tissue after treatment showed that pathologic response residuals were less than 40%. Scale bars, 500 μm. (G) Waterfall plot illustrating the cell viability based on PTC drug-testing assay and the clinical outcomes of prospective treatment among CR/PR, SD, and PD groups and corresponding ROC curve. (H and I) The drug-response profile of PTCs from (H) patient RMA-P-4513 (*n* = 3, data are represented as means ± STD) with PR and (I) patient UPS-P-8040 (*n* = 3, data are represented as means ± STD) with PD. Selected CT images are shown before and after the identified treatments.

We next validated the consistency between cell viability in PTCs and the real clinical responses of the patients in a sarcoma validation cohort. Ethical approval from the Peking University Cancer Hospital Ethics Committee was obtained, and 47 eligible patients with clinical stage III/IV sarcomas were either prospectively or retrospectively enrolled for preclinical evaluation (Figures 5A and 5B). Baseline feature information for the enrolled samples is comprehensively presented in Table S5 and Figure S5. The PTC tests and patient treatments were conducted independently, as detailed in the STAR Methods section.

A total of 60 samples were analyzed, with 8 evaluated as CR/PR, 40 as stable disease (SD), and 12 as PD following chemotherapy (Figure S5A). The accuracy in distinguishing between the CR/PR and PD groups was high at 100% for all the treatments (20/20, Wilcoxon’s test, *p* = 2.12 × 10^−4^) (Figures 5C and S5B). The efficacy of the PTC drug-testing assay in discerning clinically sensitive and resistant patients was further evaluated through receiver operating characteristic (ROC) analysis, with a calculated area under the ROC curve (AUC_ROC_) of 1.0 for the CR/PR and PD groups (Figure 5D), demonstrating exceptional accuracy.

The AUC_ROC_ for the CR/PR and SD/PD groups was 0.899, with a 95% confidence interval ranging from 80.6% to 99.2%, indicating a high level of accuracy in distinguishing between these groups (Figure 5E). Among the 40 SD samples, three samples, including MPNST-P1-2993, ES-S1-1450, and UPS-P-5880, had cell viability values of 0.36, 0.05, and 0.53, respectively (Figures 5F, S5B, and S5C). Histological examination revealed partial pathological responses with less than 40%, 50%, and 40% viable tumor cells, respectively, confirming consistency with the PTC assays (Figures 5F, S5B, and S5C). The overall accuracy in distinguishing between the CR/PR and SD/PD groups was 78.3% for all the treatments (47/60, Wilcoxon’s test, *p* = 3.19 × 10^−4^) (Figure S5D).

In case of 33 prospective treatments (Figure S5E), the AUC_ROC_ for the CR/PR and SD/PD groups was 0.864 (with a 95% confidence interval ranging from 71.47% to 100.00%) (Figures 5G and S5E), indicating consistent results observed between PTC drug testing and clinical outcomes. For instance, patient RMS-P-4513 exhibited a notable decrease (PR) in tumor size and a corresponding decrease in cell viability (0.16), while UPS-P-8040 showed an increase (PD) in tumor size and a corresponding increase in cell viability (7.15) (Figures 5H and 5I and Table S5).

### PTC prospectively identifying an effective but non-conventional therapy for an SS patient

**Figure 6:**
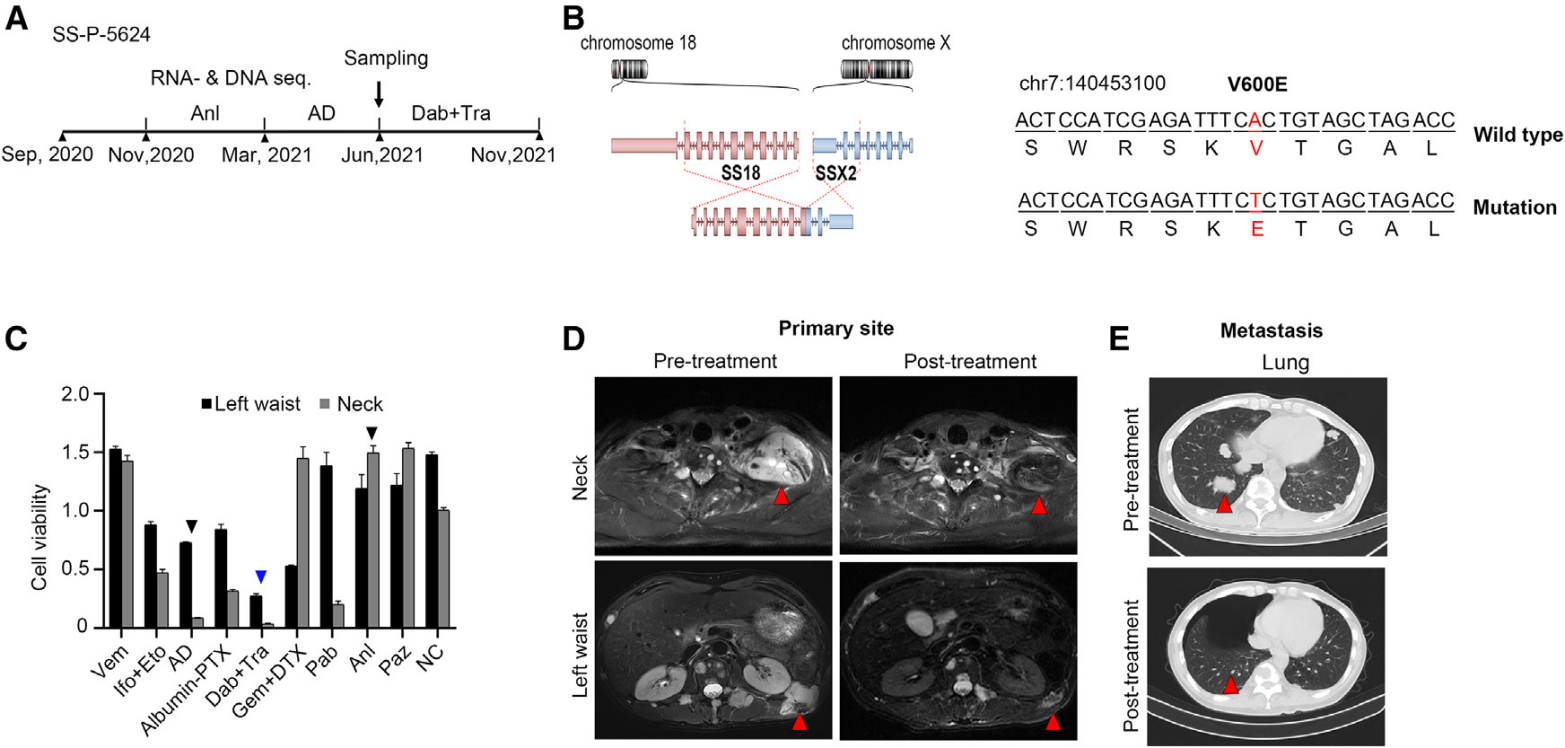
PTC prospectively identifying an effective but non-conventional therapy for a patient with SS (A) SS-P-5624 patient’s treatment history and sampling time. (B) *SS18-SSX2* fusion and *BRAF* p.V600E mutation were detected in SS-P-5624. (C) The drug-response profile of PTCs (*n* = 3, data are represented as means ± STD). (D and E) Selected CT images before and after the identified treatments in the neck (D, top), left waist (D, bottom), and lung (E).

We next implemented PTC to guide chemotherapy selection for a sarcoma patient, SS-P-5624, who faced recurrence with metastases after a conventional therapy (Figure 6A). Diagnosed with graded G2 SS in September 2020, the patient underwent immunohistochemical staining and *SYT* FISH detection confirming monophasic SS.

Prior to sampling, the patient received 5 cycles of anlotinib (Anl) and four cycles of the combination of liposome doxorubicin and dacarbazine (AD regimen) (Figure 6A). The positron emission tomography/computed tomography (PET/CT) data examined residual tumors in the neck, multiple metastases in the axial bone and bone marrow of the extremities, and lung metastases, indicating PD and SD for these two retrospective treatments (Figure 6A). Biopsies from the left lumbar and neck were provided for PTC culture and drug testing, accompanied by RNA-seq for fusion transcript detection and DNA-seq for single nucleotide mutation detection.

RNA-seq analysis identified *SS18-SSX2* fusion, while DNA-seq revealed the presence of rare BRAF p.V600E mutation (Figure 6B). This mutation is more commonly associated with hairy cell leukemia, melanomas, papillary thyroid cancers, ovarian cancers, multiple myeloma, cholangiocarcinomas, and non-small cell lung cancers (NSCLCs).^42^^,^^43^ This rarity of this mutation in SS posed two challenging questions for the personalized medicine: whether BRAF inhibitors (including vemurafenib [Vem] and dabrafenib), known for efficacy in BRAFV600E melanoma, could be applied to treat SS and which inhibitor should be chosen.^44^^,^^45^^,^^46^

The PTC drug-testing assay showed cell viability values of 1.2 (left lumbar) and 1.4 (neck) for Anl and 0.8 (left lumbar) and 0.1 (neck) for AD regimen, consistent with the retrospective clinical outcomes. Interestingly, the BRAF inhibitor Vem displayed cell viability values of 1.53 (left waist) and 1.43 (neck), while the combination of dabrafenib and trametinib (Dab + Tra) showed values of 0.27 (left waist) and 0.04 (neck) (Figure 6C). Based on these results and discussions with clinicians, the patient started dabrafenib at 150 mg orally twice a day plus trametinib at 2 mg once a day in June 2021. Subsequent imaging showed significant lesion reduction or disappearance in the neck, left waist, and lung, indicating a PR (Figures 6D and 6E).

## Discussion

With the aim of personalized medicine, we have successfully developed a Matrigel-free and serum-free culture system to generate PTCs from various sarcoma types. Notably, these PTCs not only faithfully replicate the molecular features, cell components, and pathological morphology of the original tumors but also empower us to conduct hundreds of individualized drug tests within a remarkably short time frame of just 10 days. This work has established well-defined culture conditions for PTCs, characterized by a plateau period lasting from 5 to 25 days, alongside fixed drug efficacy concentrations and a clear efficiency cutoff for drug-testing assays. These properties are paramount for establishing an effective preclinical model that facilitates the accurate prediction of clinical responses in sarcomas. As a result, PTCs have demonstrated an exceptional ability to predict patients’ clinical responses to targeted agents or chemotherapies, achieving an impressive accuracy rate of 100% in discriminating between CR/PR and PD. This presents a remarkable opportunity for STS patients to receive tailored and effective treatments, marking a significant advancement in the realm of personalized medicine.

Recent advances in omics technologies and bioinformatics have significantly deepened our understanding of sarcoma development, opening avenues for biomarker-based drug selection approaches.^47^ Several ongoing clinical trials are evaluating the efficacy of chemotherapy and immunotherapy based on gene expression patterns.^40^^,^^48^ However, the complex biological heterogeneity of sarcomas poses significant challenges on these biomarker-based drug selection approaches. Certain subtypes, such as epithelioid sarcomas with *SMARCB1* mutations, exhibit low ORRs to targeted therapies, such as the 15% response rate observed with tazemetostat. Additionally, UPS and SS, accounting for 15%–20% and 8%–10% of sarcomas, respectively, are particularly challenging to predict in terms of drug sensitivity in clinical trials. These results highlight the urgent need for new personalized medicine approaches. We propose that PTCs, which demonstrate an overall predictive accuracy of 78.3% for all clinical outcomes and 100% accuracy distinguishing CR/PR from PD, could serve as a valuable tool for personalized medicine. Integration of PTCs with biomarker-based drug selection strategies holds promise for improving treatment outcomes in sarcoma patients, as illustrated in Figure 6.

While immune checkpoint inhibitors (ICBs) have shown significant efficacy in various cancers, their impact on sarcomas has been limited. Interestingly, PTCs from sarcomas exhibit consistent immune infiltration patterns compared to the original tumors. Incorporating personalized immune cells in PTCs has the potential to replicate the immune microenvironment of each patient, providing a unique opportunity to investigate immune surveillance mechanisms in sarcomas and develop ICBs specifically targeted against these tumors.

In addition, the PTC assay offers several advantages compared to other *in vitro* tumor models. Determining the effective concentration (Ec) and cutoff for a tested drug in an *in vitro* personalized drug-testing model remains a critical yet challenging task. Traditionally, a dose-response curve is generated using parameters such as half maximal inhibitory concentration (IC_50_) for biostatistical assessment of outcomes.^20^^,^^21^ However, these measures often do not align with real-world clinical evaluation scenarios. In our study, we established a cutoff value and defined the Ec of a drug as the concentration at which the efficacy ratio (ER) in the PTC assay closely matched the ORR reported in relevant clinical trials (see Figure 4B). Additionally, PTCs can be utilized to evaluate the activity of immunotherapies.^24^ In contrast, Voabil et al. developed a patient-derived tumor fragment platform to investigate the early immunological response of human tumor tissues to *ex vivo* PD-1 blockade.^49^ However, this approach has notable limitations, including the impact of tumor heterogeneity leading to inconsistent drug responses across different wells and constraints on the number of drug assays that can be performed. Due to the significant advantages of PTCs in clinical applications summarized earlier, we have successfully launched seven investigator-initiated clinical trials (NCT05424692, NCT04130750, NCT05473923, NCT05767528, ChiCTR2000040996, ChiCTR2200061042, and ChiCTR2100048791). Several of them have already demonstrated promising results and will be submitted for publication soon.

However, the PTC model has some limitations. Prolonged culture and passage of PTCs may lead to the loss of stromal cells and immune cells, potentially altering drug-response patterns after three passages. Another limitation is the inability of PTCs to test the response to angiogenesis-based or prodrug therapies due to the absence of endothelial cells and liver cells in the model. Additionally, the small sample size in our study emphasizes the need for large-scale clinical trials to validate the clinical applicability of PTCs in personalized medicine for sarcomas.

### Limitations of the study

We have used a culture medium with the same composition across multiple sarcoma subtypes. While we have addressed the dominant subtypes, there may be differences that require further optimization to enhance culture success and improve drug-prediction accuracy. Sarcoma responses to drugs are complex and vary among patients. Although PTCs can accurately differentiate between CR/PR and PD, further improvements are needed for SD patients. To address this, we plan to expand the clinical trial to assess the effectiveness of PTCs in distinguishing SD in a larger patient cohort. Additionally, we aim to integrate more detailed pathological information to evaluate PTC-based outcomes more precisely. Furthermore, we will leverage PTCs for large-scale drug testing and explore off-label or compassionate use of drugs, offering more therapeutic possibilities for patients.

## Resource availability

### Lead contact

Further information and requests for resources and reagents should be directed to and will be fulfilled by the lead contact, Jianzhong Jeff Xi (jzxi@pku.edu.cn).

### Materials availability

Reagents used in this study are available upon reasonable request with material transfer agreement (MTA). There are restrictions to the availability of PTCs owing to hospital’s ethical regulations.

### Data and code availability

• The raw sequencing data have been deposited in Genome Sequence Archive for human of National Genomics Data Center (GSA-human of NGDC) and are publicly available as of the date of publication. Accession numbers are listed in the key resources table. The data of sequencing can be downloaded and viewed via the following link: https://ngdc.cncb.ac.cn/gsa-human/.
• This paper does not report original code.
• Any additional information required to reanalyze the data reported in this paper is available from the lead contact upon request.

## Acknowledgments

We thank our patients for giving consent and thereby allowing us access to their tissues for use in this study. We thank the National Center for Protein Sciences at Peking University in Beijing, China, and High-Performance Computing Platform at Peking University for confocal microscopy, flow cytometry, scRNA-seq, and computing resource. This work was supported in part by Basic Science Center Program of the National Natural Science Foundation of China (no. T2288102 to J.J.X.), 10.13039/501100001809National Natural Science Foundation of China (82150005 and 81827809 to J.J.X. and 82372885 to B.Y.), National Key R&D Program of China (2024YFF0507401), National Key Basic Research Project of China (2018YFA0108101 to J.J.X.), and PKU-Baidu Fund (2020BD018 to J.J.X.).

## Author contributions

PTC cultures: S.Y. and J.J.X. Sequencing and analysis of sequencing data: X. He and J.W. Immunofluorescent staining and flow cytometry: X. He, X. Hu, and Y.W. Surgery: T.G., J. Liu, C.B., Y.S., F.C., S.L., Y.-J.S., R.X., L.Z., Z.F., X.W., M.L., W.F., and Z.F. Clinical outcome evaluation: Z.T., F.C., and L.T. DNA/RNA sequencing libraries: Juan Li, Y.H., Jiaxin Li, H.L., and H.Z. Funding acquisition: J.J.X. and B.Y. Project administration: J.J.X. and Z.F. Supervision: J.J.X. and Z.F. Writing – original draft: X. He, J.W., S.Y., B.Y., and J.J.X. Writing – review and editing: J.J.X. and Z.F.

## Declaration of interests

S.Y. and H.Z. are employees and shareholders of GeneX Health Co., Ltd. (GeneX Health), and J.J.X. is a shareholder of GeneX Health. GeneX Health has submitted one patent regarding PTC culture (A method for the culture of primary lung cancer cells, ZL 202111135387.8).

## STAR★Methods

### Key resources table

**Table:** 

REAGENT or RESOURCE	SOURCE	IDENTIFIER
**Antibodies**		

anti-human CD45	Biolegend	Cat#368509; RRID:AB_2566369
anti-human Vimentin	R&D SYSTEMS	Cat#IC2105R; RRID:AB_3654985
anti-human Pan Cytokeratin	Invitrogen	Cat#53-9003-80; RRID:AB_1834351
anti-human CD3	Biolegend	Cat# 300434; RRID:AB_10962690
anti-human CD68	Invitrogen	Cat# 12-0689-41; RRID:AB_10804042
anti-human CD11c	Biolegend	Cat# 301613; RRID:AB_493024
anti-human CD20	Biolegend	Cat# 980202; RRID:AB_2616617
anti-human S100A9	Biolegend	Cat# 350705; RRID:AB_2564007
anti-human CD326	Biolegend	Cat# 324204; RRID:AB_756078
Anti-human TEF3	Abclonal	A0548; RRID:AB_2861464
Anti-human S100	Zhong Shan Jin Qiao	ZA-0225; RRID:AB_2924228
Anti-human CD34	Zhong Shan Jin Qiao	ZM-0046; RRID:AB_2924244
Anti-human INI1	Zhong Shan Jin Qiao	ZM-0173; RRID: AB_3676391
Anti-human VIM(Vimentin)	Zhong Shan Jin Qiao	ZM-0260; RRID:AB_2864331
Anti-human Bcl2	Abcam	AB32124; RRID:AB_725644
Anti-human TLE1	Abclonal	A3528; RRID:AB_2863078
Anti-human STAT6	Abcam	AB32520; RRID:AB_778113
Anti-human CD34	Zhong Shan Jin Qiao	ZM-0046; RRID:AB_2924244
Anti-human CK	Zhong Shan Jin Qiao	ZM-0069; RRID:AB_2941997
Anti-human MDM2	Invitrogen	337100; RRID:AB_2533136
Anti-human CDK4	Zhong Shan Jin Qiao	ZA-0614; RRID:AB_3676245
Anti-human Desmin	Zhong Shan Jin Qiao	ZA-0610; RRID: AB_3676388
Anti-human MUC4	Abcam	AB150381; RRID: AB_3676389
Anti-human EMA	Zhong Shan Jin Qiao	ZM-0095; RRID: AB_3676390

**Biological samples**		

Human sarcoma tissue	Peking University Cancer Hospital	N/A

**Chemicals, peptides, and recombinant proteins**		

MSP	SGE biotechnology	MST-908
HGF	Peprotech	100–39
EGF	Peprotech	AF-100-15
FGF-basic	Peprotech	100-AF-18B
N-acetyl-L-cysteine	Sigma	A9165
Y-27632	Selleck	S6390
Citrate Unmasking Solution	Cell Signaling Technology	14746
DAB	Cell Signaling Technology	8059
Paraformaldehyde	Sigma-Aldrich	P6148
M2 medium	Macgene	CE003
Bovine serum albumin	Meijing	A738328
Trizol	Thermo	15596026
Chloroform	Sigma-Aldrich	67-66-3
Isopropanol	Sigma-Aldrich	67-63-0
Hyaluronidase	SIGMA	H3506-1G
Cell Staining Buffer	Biolegend	420201
Human TruStain FcX™	Biolegend	422302
IL-2	Peprotech	AF-200-02
IL-7	Peprotech	AF-200-07
IL-15	Peprotech	AF-200-15
Cisplatin	Selleck	S1166
5-FU	Selleck	S1209
Oxaliplatin	Selleck	S1224
Paclitaxel	Selleck	S1150
Docetaxel	Selleck	S1148
Irinotecan	Selleck	S1198
Raltitrexed	Selleck	S1192
Doxorubicin HCl	Selleck	S1208
Epirubicin HCl	Selleck	S1223
Etoposide	Selleck	S1225
Mitomycin C	Selleck	S8146
Regorafenib	Selleck	S1178
Gefitinib	Selleck	S1205
Carboplatin	Selleck	S1215
Gemcitabine	Selleck	S1714
Olaparib	Selleck	S1060
Vinorelbine Tartrate	Selleck	S4269
Tamoxifen	Selleck	S1238
Fulvestrant	Selleck	S1191
Afatinib	Selleck	S1011
Osimertinib	Selleck	S7297
Sunitinib	Selleck	S7781
Crizotinib	Selleck	S1068
Everolimus	Selleck	S1120
Palbociclib	Selleck	S4482
Pirarubicin	Selleck	S1393
Lapatinib	Selleck	S2111
Neratinib	Selleck	S2150
Ceritinib	Selleck	S7083
Pemetrexed	Selleck	S5971
Erlotinib HCl	Selleck	S1023
Icotinib	Selleck	S2922
Nedaplatin	Selleck	S1826
Vemurafenib	Selleck	S1267
Ifosfamide	Selleck	S1302
Dabrafenib	Selleck	S2807
Carmustine	Selleck	S3669
Bleomycin sulfate	Selleck	S1214
Dacarbazine	Selleck	S1221
Trametinib	Selleck	S2673
Cobimetinib	Selleck	S8041
Ponatinib	Selleck	S1490
Imatinib	Selleck	S2475
Nab-Paclitaxel	Selleck	E1068
Cabozantinib	Selleck	S1119
Lenalidomide	Selleck	S1029
Sorafenib	Selleck	S7397
Penicillin-streptomycin	Thermo Fisher	BP2959-50
GlutaMAX	Thermo Fisher	35050061
B27	Thermo Fisher	A1895601
ITS-X	Thermo Fisher	I917636
MEM Non-Essential Amino Acid Solution	Thermo Fisher	11140050

**Critical commercial assays**		

EdU imaging kits Alexa Flour 594	Beyotime	C0078L
DNeasy Blood & Tissue Kit	Qiagen	69504
NEBNext Ultra II DNA library Prep Kit	New England Biolabs	E7103
NEBNext Ultra II RNA library Prep Kit for Illumina	New England Biolabs	E7770
ZytoLight SPEC SPEC TFE3 Dual Color Break Apart Probe	ZytoVision	Z-2109-50

**Deposited data**		

DNA TMB seq data, WGS seq data	This paper	GSA-human PRJCA024849
single-cell RNA-seq data	This paper	GSA-human PRJCA024849
Bulk PTC and Tumor RNA expression matrix	This paper	GSA-human PRJCA024849

**Software and algorithms**		

FlowJo_V10.8.1	FlowJo LLC	https://www.flowjo.com/
Prism version 9	GraphPad	https://www.graphpad.com/
ImageJ	Schneider et al.^50^	https://imagej.net
HALO	Indicalab	https://indicalab.com/halo/
GATK v4.1.9	McKenna et al.^51^	https://github.com/broadinstitute/gatk
BWA v0.7.17	Li^52^	https://github.com/lh3/bwa
Picard v2.20.5	Broad Institute	https://github.com/broadinstitute/picard
IGV v2.8.9	Robinson et al.^53^	https://github.com/igvteam
Variant Effect Predictor v102	McLaren et al.^54^	https://github.com/Ensembl/ensembl-vep
snpEff/snpSift	Cingolani et al.^55^	https://pcingola.github.io/SnpEff/
trim_galore	Krueger et al.^56^	https://github.com/FelixKrueger/TrimGalore
vcf2maf	Krueger et al.^56^	https://github.com/mskcc/vcf2maf
maftools R package v2.6.0	Mayakonda et al.^57^	https://bioconductor.org/packages/release/bioc/vignettes/maftools/inst/doc/maftools.html
CNVkit	Talevich et al.^58^	https://github.com/etal/cnvkit
DESeq2	Love et al.^59^	https://bioconductor.org/packages/release/bioc/html/DESeq2.html
Salmon V1.2.1	Patro et al.^60^	https://github.com/COMBINE-lab/salmon
MCPcounter R package	Becht et al.^39^	https://github.com/ebecht/MCPcounter
xCell	Aran et al.^41^	https://github.com/dviraran/xCell
tximport R package	Soneson et al.^61^	https://github.com/thelovelab/tximport
Arriba v2.0.0	Uhrig et al.^62^	https://github.com/suhrig/arriba
Cell Ranger v5.0.1	10x Genomics	https://www.10xgenomics.com/support/software/cell-ranger/downloads
Scanpy v1.7.1	Wolf et al.^63^	https://github.com/scverse/scanpy
Scrublet	Wolock et al.^64^	https://github.com/swolock/scrublet
Harmony	Korsunsky et al.^65^	https://github.com/immunogenomics/harmony
Python	Python Software Foundation	https://www.python.org
R v. 4.2.1	The R Foundation	https://www.r-project.org
RStudio	Posit Software	https://posit.com
ggplot2	Wickham^66^	https://ggplot2.tidyverse.org/
Analysis code	This paper	This paper does not report original code

### Experimental model and study participant details

#### Human samples

All human tissue samples were obtained from Peking University Cancer Hospital. Before performing the procedure, all patients provided written informed consent to allow the use of excess tissue for the study. The study was approved by the Ethics Committee. The pathologic status of the specimens was provided by the hospital. PTCs, clinical outcome, and safety data on a per-patient level can be obtained through the Institutional Review Board of the Peking University and patients were informed consent. Detailed patient clinicopathological characteristics including age, gender, tumor staging and grading histology, therapy and clinical outcome were outlined in Table S5.

### Method details

#### Study design and patients

This is an observational trial. The aim of the study was to assess the feasibility and predictive value of a standardized PTC-based test to differentiate efficacy of the patients' clinical drug regimens. The study was conducted at Peking University Cancer Hospital and was approved by the local ethical review board. The study protocol was in accordance with the Declaration of Helsinki, Chinese law and Good Clinical Practice. Clinical response of biopsied lesions was assessed by radiologists and internists according to RECIST criteria. Written informed consent was obtained from patients and/or their authorized representatives.

Patients who are able to successfully construct an *in vitro* drug sensitivity model of PTC and are planning preoperative neoadjuvant chemotherapy or chemotherapy for advanced unresectable soft tissue sarcoma are enrolled. Subjects who met the enrollment criteria were treated with chemotherapy regimens in accordance with the recommendations of the Chinese Society of Clinical Oncology (CSCO) guidelines for the diagnosis and treatment of soft tissue sarcoma, taking into account the subjects' general condition, tumor site, pathologic staging, and other clinical factors. Tumor assessment was performed periodically, and the drug was administered continuously until the patient experienced an intolerable toxic reaction that did not resolve after dose adjustment, or until the subject experienced disease progression according to RECIST 1.1, or until the subject met other withdrawal criteria. Clinical efficacy will be evaluated by the clinician in charge and the multidisciplinary team, both of which are independent of each other.

The study subjects of this trial are patients who have undergone preoperative neoadjuvant chemotherapy or chemotherapy for advanced unresectable soft tissue sarcoma from 2019 to 2022. Inclusion criteria included voluntary enrollment and signed informed consent, age 18 and 75 years, soft tissue sarcoma diagnosed by pathohistology or cytology, at least one measurable target lesion according to RECIST 1.1, expected survival ≥3 months, ECOG physical status score ≤2, and those who might be able to obtain a fresh specimen by puncture or surgical means. Other exclusion criteria included failure of PTC *in vitro* drug sensitization modeling, severe organ dysfunction, pregnant or lactating females, cognitive dysfunction or psychiatric disorders, and a history of other illnesses affecting the test. Exclusion criteria included inability to continue treatment due to epidemics or other factors, trial or unavailability of examination data, and patient withdrawal. Subjects could opt out of the study at any time for any reason.

Based on the patient’s condition, the doctor recommends off-label treatment. Under *the Implementation Rules of the Regulations for the Administration of Medical Institutions*, treatment should strictly adhere to established indications. However, given the patient’s unsatisfactory response to the current medication regimen within the prescribed guidelines, the doctor proposes an off-label treatment to optimize therapeutic outcomes. After carefully considering potential adverse reactions, contraindications, and precautions, and drawing on clinical experience, it is determined that the potential benefits of the alternative treatment plan outweigh the possible risks. This drug is not part of a clinical trial or scientific research, but its clinical use has been shown to provide more benefits than harm. Informed consent has been obtained from the patient.

#### Experimental model and subject details

The patients and corresponding PTCs were divided into three sets: characterization and storage set, assay set and validation set. The characterization and storage set was used to characterize PTCs in comparison with original tumor samples or stored for future study. The assay set was separated into two groups to determine the drug efficacy concentration of a targeted therapy or chemotherapy due to their different action mechanisms. The validation set was used to compare the consistency between PTC drug assays and clinical outcome.

##### Characterization and storage set

Of 254 PTCs, 136 were either thoroughly characterized in comparison with original tumor samples or preserved for future analysis.

##### Assay set

The assay set used in our training study consisted of 135 PTCs. This set was used to experiment with 25 common chemotherapeutic drugs, with 3–6 concentration gradients set for each drug. Within the training cohort, we established drug effects and cell viability cutoffs, in addition to determining the drug Ec, with reference to clinical evaluation scenarios (Table S4).

##### Validation set

In this validation set, a total of 56 sarcoma patients were enrolled from Peking University Cancer Hospital with 37 for prospective study and 19 for retrospective study separately. However, 3 were excluded due to PTC culturing failure and 4 were excluded due to regimen adjustments not in line with the study design in prospective study. In retrospective study, 2 were excluded due to PTC culturing failure.

Among the remaining 47 patients, 33 samples had prospective clinical outcomes, and 27 samples had retrospective clinical outcomes. These patients underwent standardized chemotherapy or targeted therapy following updated NCCN guidelines and clinical evaluations. Procedures included CT scans or MRI examinations every 2–3 treatment cycles and efficacy evaluations scored using RECIST 1.1 by two independent researchers. Corresponding PTCs were subjected to drug testing within 2 weeks after sampling. Notably, no information about PTC test results was disclosed to clinicians or patients before or during treatment. Comprehensive patient clinicopathological characteristics are outlined in Table S5.

#### Patient treatment

Patients received standardized chemotherapy or targeted therapy following the updated NCCN guidelines. According to different pathological types, patients received different standard antitumor treatments.

For patients without sensitive mutations, paclitaxel combined with Liposome doxorubicin combined with Ifosfamide (AI) or Liposome doxorubicin combined with Dacarbazine (AD) or Gemcitabine combined with Docetaxel (GD) is often used. Liposome doxorubicin recommended dose of 30-40 mg/m^2^ intravenously drip over 2 days and ifosfamide recommended dose of 1.8–2 g/m^2^ intravenously drip on day 1 to day 5, repeated every 3 weeks. Liposome doxorubicin recommended dose of 30–40 mg/m^2^, dacarbazine recommended dose of 0.9–1 g/m^2^ and intravenously drip every 3 weeks. Gemcitabine recommended dose of 1 g/m^2^ intravenously drip on day 1 and day 8 and docetaxel recommended dose of 75 mg/m^2^ intravenously drip on day 2, every 3 weeks. Vincristine recommended dose of 1–2 mg/m^2^ (1.4 mg/m^2^, max 2 mg; age >65 max 1 mg), liposome doxorubicin recommended dose of 30–40 mg/m^2^ and cyclophosphamide recommended dose of 1 g/m^2^ and intravenously drip every 3 weeks. Vincristine recommended dose of 1–2 mg/m^2^ (1.4 mg/m^2^, max 2 mg; age >65 max 1 mg) on day 1, liposome doxorubicin recommended dose of 30–40 mg/m^2^ on day 1 and Ifosfamide recommended dose of 1.8–2 g/m^2^ intravenously drip on day 1 and day 5, every 3 weeks. Vincristine recommended dose of 1–2 mg/m^2^ (1.4 mg/m^2^, max 2 mg; age >65 max 1mg), liposome doxorubicin recommended dose of 30–40 mg/m^2^ and ifosfamide recommended dose of 1.8–2 g/m^2^ intravenously drip on day 1 and day 5, and etoposide recommended dose of 100 mg/m^2^ day 1 to day 5, every 3 weeks. Crizotinib recommended dose of 250 mg orally twice daily. Anlotinib recommended dose of 8–12 mg orally daily on day 1 to day 14 and then every 3 weeks, and everolimus recommended dose of 10 mg orally daily or anlotinib recommended dose of 8–12 mg orally daily on day 1 to day 14 and then every 3 weeks alone. Albumin paclitaxel (nab-PTX) recommended dose of 260 g/m^2^ intravenously drip on day 1, and then every 3 weeks. Darafenib recommended dose of 150 mg orally twice daily and trametinib recommended dose of 2 mg orally once daily.

#### Culture of sarcoma PTCs

Collected fresh samples were conditioned in ice-cold PBS with 10 mM HEPES and 100 U/mL penicillin-streptomycin (Thermo Fisher Scientific). Necrotic areas and adipose tissue were removed as possible. Tissues were minced into small pieces and digested in 5 mL PBS/EDTA 1 mM containing collagenase I (Thermo Fisher Scientific) 200 U/mL for 1 h. 40 μm filters were used to collect dissociated cells. After 10 min’ centrifugation (300 × g, 4°C), cell pellets were re-suspended in PTC growth medium (Table S1) and seeded on a multi-well plate (GeneX Health, GX-07) at the concentration of 10^5^ cells/cm^2^. Cells were cultured in incubator at 37°C, 5% CO_2_. PTC growth medium was refreshed every 2–3 days, as necessary.

#### Culture of sphere

The serum-free sphere culture medium was adjusted according to previously reported literature^67^ and consisted of DMEM/F12 (1:1) supplemented with 4% B27 (reagents from Invitrogen), 20 ng/mL recombinant human epidermal growth factor (rhEGF; Strathmann Biotech, Hamburg, Germany), 20 ng/mL leukemia inhibitory factor (LIF), and 10 IU/mL (5 μg/mL) heparin (Roche, Mannheim, Germany). We replaced L-glutamine with GlutaMAX dipeptide to prevent degradation of glutamine and ammonia accumulation in long-term cultures. Samples were cultured in ultra-low attachment 24-well plates (Costar, Corning, NY, USA) and maintained in an incubator at 37°C with 5% (vol/vol) CO_2_.

#### Histology of PTCs and parental tumor tissues

PTCs were collected by centrifugation at 300 × g and 4°C for 10 min. Tissues and PTCs were washed with cold PBS, and fixed in PBS containing 4% paraformaldehyde overnight. The pellets were paraffin-embedded, and 5-μm-thick paraffin sections were generated. The PTC or tissue morphology was determined by H&E staining. The H&E images were obtained using a Nikon Ti2-U microscope.

#### Immunohistochemistry of PTCs and tumor tissues

Paraffin sections were dewaxed and rehydrated through a graded ethanol series. After heat-mediated antigen retrieval with Citrate Unmasking Solution (Cell Signaling Technology, 14746), the sections were blocked at room temperature for 1 h. The primary antibodies were diluted in 3% BSA, and staining was performed overnight at 4°C with gentle rocking. DAB (Cell Signaling Technology, 8059) was used to provide an acceptable staining intensity. The stained tissue was visualized using the Nikon Ti2-U microscope.

#### Immunofluorescence staining

PTCs were collected and fixed with 4% paraformaldehyde (Sigma-Aldrich, P6148) for 20 min followed by washing with M2 medium (Macgene, CE003) with 0.5% bovine serum albumin (Meijing, A738328). PTCs were permeabilized with 1% Triton X-100 in PBS for 30 min, and blocked with 10% goat serum for 30 min. Tissue and PTCs were stained with fluorescent conjugated primary antibodies. Antibodies used in the assays were listed in Table S6. Images were obtained with Nikon Eclipse Ti2 laser scanning confocal microscopy (Nikon). The software ImageJ was used for image analysis.

#### Flow cytometry

PTC were harvested with 40 μm filters, and digested into single cells using trypsin followed with collagenase (II and IV) and hyaluronidase (Thermo Fisher Scientific). After centrifugation, a single cell suspension was prepared in Cell Staining Buffer (Biolegend). Cells were centrifuged at 350 × g for 5 min, and pellets were resuspended in Cell Staining Buffer at 106 cells/mL. Human TruStain FcX (Biolegend) was used to block Fc receptors. Conjugated fluorescent antibodies were added and cell suspensions were incubated on ice for 15 min in the dark. Cells were washed twice with Cell Staining Buffer and then analyzed with a Flow Cytometer (BD).

#### PTC drug testing

PTCs with more than 40 μm in diameter were collected (40-μm filters, BD Falcon), centrifuged at 300 × g for 10 min, washed with PBS, and re-suspended with the NSCLC growth medium. Then, 100 μL of a medium containing 30–50 PTCs was seeded into a Teflon-modified chip (GX-01, GeneX Health). Next, 50 μL of the sarcoma PTC growth medium containing the drug was added to the chip. The chip was incubated at 37°C and 5% CO_2_. Images of each well were screened with the Nikon Ti-U microscope system.

The PTC cell viability was performed by experienced technicians who were blinded to the interventions and clinical outcomes of the patients. For a validation set of patients, their clinical responses were evaluated according to the RECIST criteria version 1.1 by radiologists and physicians based on MRI or CT scan. For each patient, 1–2 target lesions were evaluated, including the primary tumor with diameter >10 mm and lymph node short axis ≥15 mm. Other non-measurable lesions, like the bone and pleural metastasis, were not included into the calculation, although their responses were consistent with the target lesions. The data for all PTC drug tests are shown in Table S5.

#### EdU assay

Cell proliferation ability was measured by Click-it EdU imaging kits (Thermo, Alexa Flour 594). In brief, PTCs were incubated in growth medium with 20 μM EdU for 1 h. After a 4% PFA fixation step and a 0.5% Triton X-100 permeabilization step, cells were incubated in Click-iT Plus reaction cocktail for 30 min. Nuclei were marked by 5 μg/mL Hoechst 33342 overnight. Images were captured with Nikon Ti-U microscope system.

#### FISH

According to the manufacturers’ instructions, ZytoLight SPEC TFE3 Dual Color Break Apart Probe was used on formalin-fixed paraffin-embedded tumor tissues in FISH, which is a mixture of three direct labeled probes. The ZytoLight SPEC TFE3 Dual Color Break Apart Probe is composed of: ZyOrange (excitation 547 nm/emission 572 nm) labeled which target sequences mapping in Xp11.23∗ (chrX:48,287,169-48,792,674) distal to the *TFE3* and ZyGreen (excitation 503 nm/emission 528 nm) labeled which target sequences mapping in Xp11.23∗ (chrX:48,906,685-49,509,699) proximal to the *TFE3*. 4 μm thick formalin-fixed paraffin-embedded sections were deparaffinized, treated with warmed heat pretreatment solution citric at 98°C, and digested in pepsin solution. Target DNA and probes were co-denatured at 75°C for 10 min and incubated at 37°C overnight in a humidified hybridization chamber. Finally, washing slides, air dried and DAPI staining were performed. Signals for each locus specific FISH probe were assessed under LSM880 laser scanning confocal microscopy (Zeiss).

#### Bulk DNA and RNA extraction

For sample preparation, the specimens were washed twice in phosphate-buffered saline (PBS) before DNA or RNA isolation. DNA was extracted from tissues or cultured cells by proteinase K digestion in combination with the DNeasy Blood & Tissue Kit (Qiagen), following the manufacturer’s protocol. RNA was isolated using Trizol method. Each sample was resuspended in 500 μL Trizol (Thermo Fisher Scientific), briefly vortexed, and 100 μL chloroform (Sigma-Aldrich) was added. Phase separation was achieved by centrifuging the sample at 12,000 × g for 15 min at 4°C, then 200 μL of the aqueous phase was carefully transferred to a new tube. The RNA precipitation was carried out with 200 μL isopropanol (Sigma-Aldrich) and the RNA pellet was washed with 1 mL of 70% ice-cold ethanol.

#### Bulk DNA and RNA library preparation and sequencing

Targeted library preparation and sequencing were outsourced to GX-Health Corporation. In brief, whole genomic DNA libraries were prepared using NEBNext Ultra II DNA library Prep Kit for Illumina (New England Biolabs), the protocol consists of several enzymatic and purification steps. Targeted DNA sequencing libraries were generated with a TMB panel that targets 333 cancer-related genes in Table S2. RNA libraries were prepared by NEBNext Ultra II RNA library Prep Kit for Illumina (New England Biolabs). Libraries were analyzed by Bioanalyzer 2100 and DNA 1000 or DNA high-sensitivity chips. Libraries were quantified using Illumina library qPCR quantification kits from KAPA Biosystems. Paired-end sequencing (2 × 150 bp) was performed with Illumina HiSeq Xten.

#### Genomic mutation and copy number analysis

Sequencing data were processed following GATK (v4.1.9) best practices.^51^ Briefly, paired-end reads were mapped to the human reference genome (hg19) using BWA mem (v0.7.17)^52^ followed by duplicates removal with Picard Mark Duplicates (v2.20.5). Excess fragments of duplicate PCR copies were removed from targeted sequencing by the Mark Duplicates algorithm. The Base Recalibrator and ApplyBQSR algorithms of GATK4 were then used to detect and correct systematic errors in the base quality of sequencing pairs during sequencing, respectively.

Somatic mutations were determined by Mutect2 algorithm for each pair of tumor tissues or microtumor models, and their corresponding paratumor tissue samples. Learn Read Orientation Model and Filter Mutect Call were used for strand and base preference correction, and filtering. Finally, The somatic variants were annotated and converted to maf format by Variant Effect Predictor (v102),^54^ snpEff^55^ and vcf2maf. Finally, graphing and further analysis were performed by R package maftools (v2.6.0).^57^

Mean depth of whole-genome sequencing was 3× (range 2×-5×). Sequencing data processing workflow was the same as above, except that base quality recalibration was not performed. Copy number analysis was performed by CNVkit^58^ under WGS mode using CBS segmentation and the average bin size was set to 5000.

#### RNA sequencing analysis

For mRNA sequencing analysis, quality-controlled paired-end data were quantified by Salmon (V1.2.1)^60^ in the mapping-based mode, using transcriptome reference GRCh38 release-96 and annotation downloaded from Ensembl. Gene expression was calculated by aggregating transcript expression (TPM) belonging to the same gene, and the TPM of the gene was then converted to read counts implemented in the R function tximport. Top 500 most variable genes were selected based on the variance among all the samples. Gene fusions were identified by Arriba (v2.0.0) with default parameters.^62^

#### Single-cell RNA library preparation and sequencing

The specimens were washed with Hanks Balanced Salt Solution (HBSS) for 3 times and minced into 1–2 mm pieces. The specimens were dissociated into single cell suspension with a Singleron PythoNTM Automated Tissue Dissociator (Singleron Biotechnologies) with sCelLiveTM Tissue Dissociation Mix (Singleron Biotechnologies), based on the preset protocol for sarcoma. The sample was stained with trypan blue (Sigma, Shanghai, China) and microscopically evaluated for cell viability. ingle-cell suspensions at 1 × 10^5^ cells/mL in concentration in PBS (HyClone, Shanghai, China) were prepared and loaded onto microfluidic devices and scRNA-seq libraries were constructed according to Singleron GEXSCOPE protocol by GEXSCOPE Single-Cell RNA Library Kit (Singleron Biotechnologies) and Singleron Matrix Automated single-cell processing system (Singleron Biotechnologies). Individual libraries were diluted to 4 ng/μL and pooled for sequencing. Pools were sequenced on Illumina novaseq6000 with 150 bp paired end reads.

#### Analysis of single-cell RNA sequencing data

Sequencing data were aligned to GRCh38 and processed by Cell Ranger (v5.0.1). The generated filtered cell UMI count matrixes were analyzed with Scanpy (v1.7.1).^63^ Each sample is quality controlled separately and merged together for downstream analysis. Briefly, cells with fewer than 500 genes expressed and with more than 15% of reads from mitochondrial genes were discarded. Potential doublets are calculated and removed by Scrublet.^64^ Highly variable genes are selected by function *scanpy.pp.highly_variable_genes* with parameter n_top_genes = 2000. Then, effects of the total count per cell, the percentage of mitochondrial gene and the cell cycle genes are regressed out by using *scanpy.pp.regress_out* function. A principal component analysis (PCA) matrix with 50 components is calculated using *scanpy.tl.pca* function with parameter “*svd_solver = 'arpack'* ”. To remove the batch effect from different donors, and integrate data from multiple experiments, *Harmony*^65^ algorithm is used by *scanpy.external.pp.harmony_integrate* function to get the adjusted principal components. A neighborhood graph is built according to the top 30 adjusted principal components by *scanpy.pp.neighbors* function with parameter “*use_rep = 'X_pca_harmony',n_pcs = 30,n_neighbors = 25.*”

The UMAP dimension reduction is calculated to visualize data by *scanpy.tl.umap* function with default parameters. To cluster cells by their expression profiles, we use an unsupervised graph-based clustering algorithm called Leiden with a resolution of 1.0. The marker genes for each cluster are identified by Wilcoxon test using *scanpy.tl.rank_genes_groups* function with parameter “*method = 'wilcoxon',pts = True.*”. Clusters with mitochondria genes as markers were removed from analysis.

### Quantification and statistical analysis

#### High-throughput drug-screening analysis

All statistical analyses were performed in R or Excel. *p* values were corrected for multiple testing using the Benjamini-Hochberg procedure to calculate the false discovery rate. The correlation between a continuous variable and an ordinal categorical variable was calculated using Spearman’s correlation.

#### Validation set analysis

We standardized all continuous measures of the cell viability by using mean and standard deviations. Groups were compared using two-sample Wilcoxon’s test (called as Mann-Whitney test). Fisher’s exact test was used to test the independence of two categorical variables.

### Additional resources

The trial was registered in the Chinese Clinical Trial Registry as ChiCTR2400085510 (https://www.chictr.org.cn/showproj.html?proj=233237) and supported by Peking University Cancer Hospital.

## Supplemental information

Document S1. Figures S1–S5 and Tables S1, S3, and S4

Table S2. Gene fusion detected in 23 pairs of PTCs and the original tumors and mutation panel genes used in WGS, related to Figure 2

Table S5. Sample clinical information and medicine information used in drug test, related to Figure 5

Document S2. Article plus supplemental information
