# Supplementary material for: Self-assembled patient-derived tumor-like cell clusters for personalized drug testing in diverse sarcomas
Source: Cell Rep Med. 2025 Mar 6;6(3):101990. doi: 10.1016/j.xcrm.2025.101990 (PMC11970405; doi:10.1016/j.xcrm.2025.101990)
Supplement: Document S1. Figures S1–S5 and Tables S1, S3, and S4 [file mmc1.pdf]

Cell Reports Medicine, Volume *6*

Supplemental information

Self-assembled patient-derived tumor-like cell clusters for personalized drug testing in diverse sarcomas

Tian Gao, Xinyu He, Junyi Wang, Jiayong Liu, Xiongbing Hu, Chujie Bai, Shenyi Yin, Yunfei Shi, Yanmin Wang, Zhichao Tan, Fang Cao, Shu Li, Yan-Jie Shi, Ruifeng Xue, Juan Li, Yang He, Jiaxin Li, Huinan Lu, Hanshuo Zhang, Lu Zhang, Zhiwei Fang, Xinyu Wang, Mengmeng Liu, Wenjun Fu, Lei Tang, Buqing Ye, Zhengfu Fan, and Jianzhong Jeff Xi

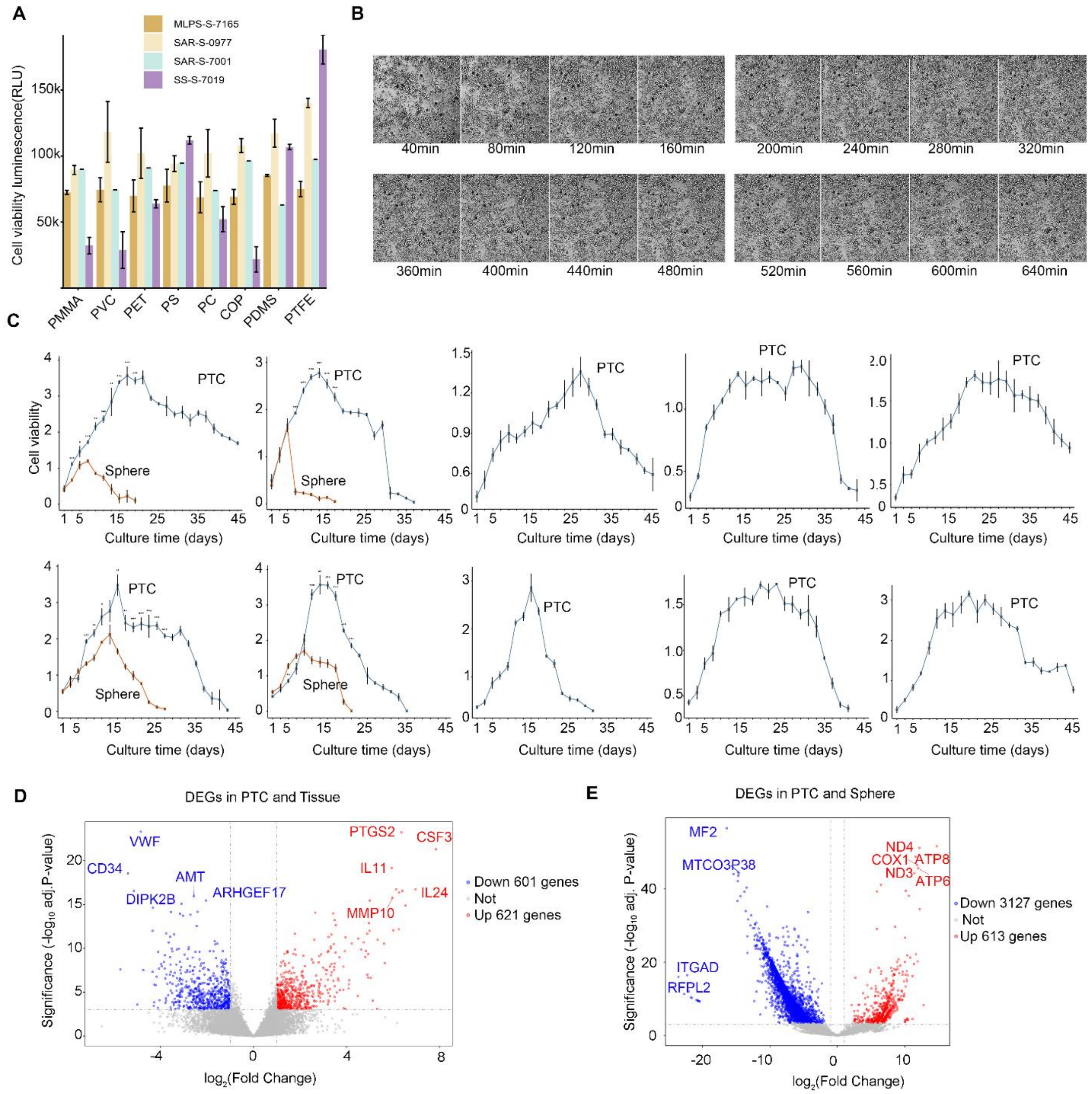

Figure S1. PTC Culture (related to Figure 1).

(**A**) Fluorescence intensity of CellTiter-Glo cell activity in different culture materials with different fractions of PTC culture (n=3, data are represented as means ± STD). PMMA, polymethyl methacrylate; PVC, Polyvinyl chloride; PET, Polyethylene terephthalate; PS, Polystyrene; PC, Polycarbonate; COP, Cycloolefin polymer; PDMS, Polydimethylsiloxane; PTFE, Polytetrafluoroethylene.

(**B**) Time course of primary cell clustering in the first 40, 80 and 120 minutes until 8 hours. Scale bar: 100 μm. Also see Movie S1.

(**C**) Growth curves of PTC and sphere from the same sample (n=3, data are represented as means ± STD).

(**D**) Volcano plot showing differential gene expression between PTCs and tumor samples (n =1,222, adjusted p-value < 0.001).

(**E**) Volcano plot showing differential gene expression between PTCs and tumor spheres (n = 3,740, adjusted p-value < 0.001).

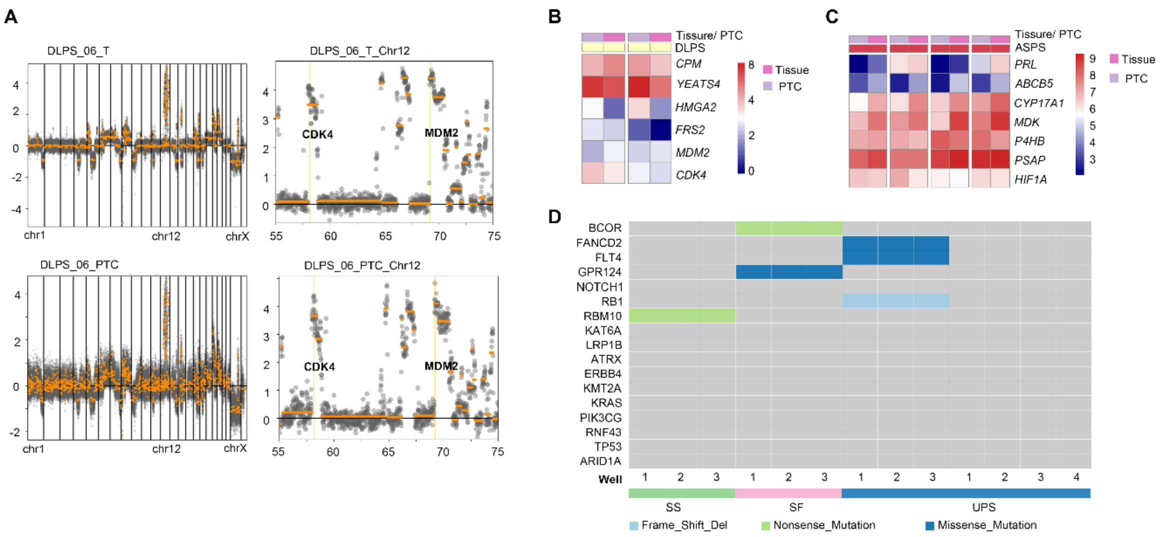

Figure S2. Genomic and Gene Expression Comparison of PTCs with the Original Tumors (related to Figure 2).

(**A**) Characteristic copy number variation of dedifferentiated liposarcoma in PTC model and primary tumor tissue, especially in chromosomes 12 *CDK4* and *MDM2*. T: tumor; P: PTC.

(**B**) Characterized genes expression in dedifferentiated liposarcomas of tissue and PTC. T: tumor; P: PTC.

(**C**) Characterized genes expression in alveolar soft tissue sarcoma of tissue and PTC. T: tumor; P: PTC.

(**D**) The somatic mutations in replicated PTCs. 0 to 4 for PTCs in different wells.

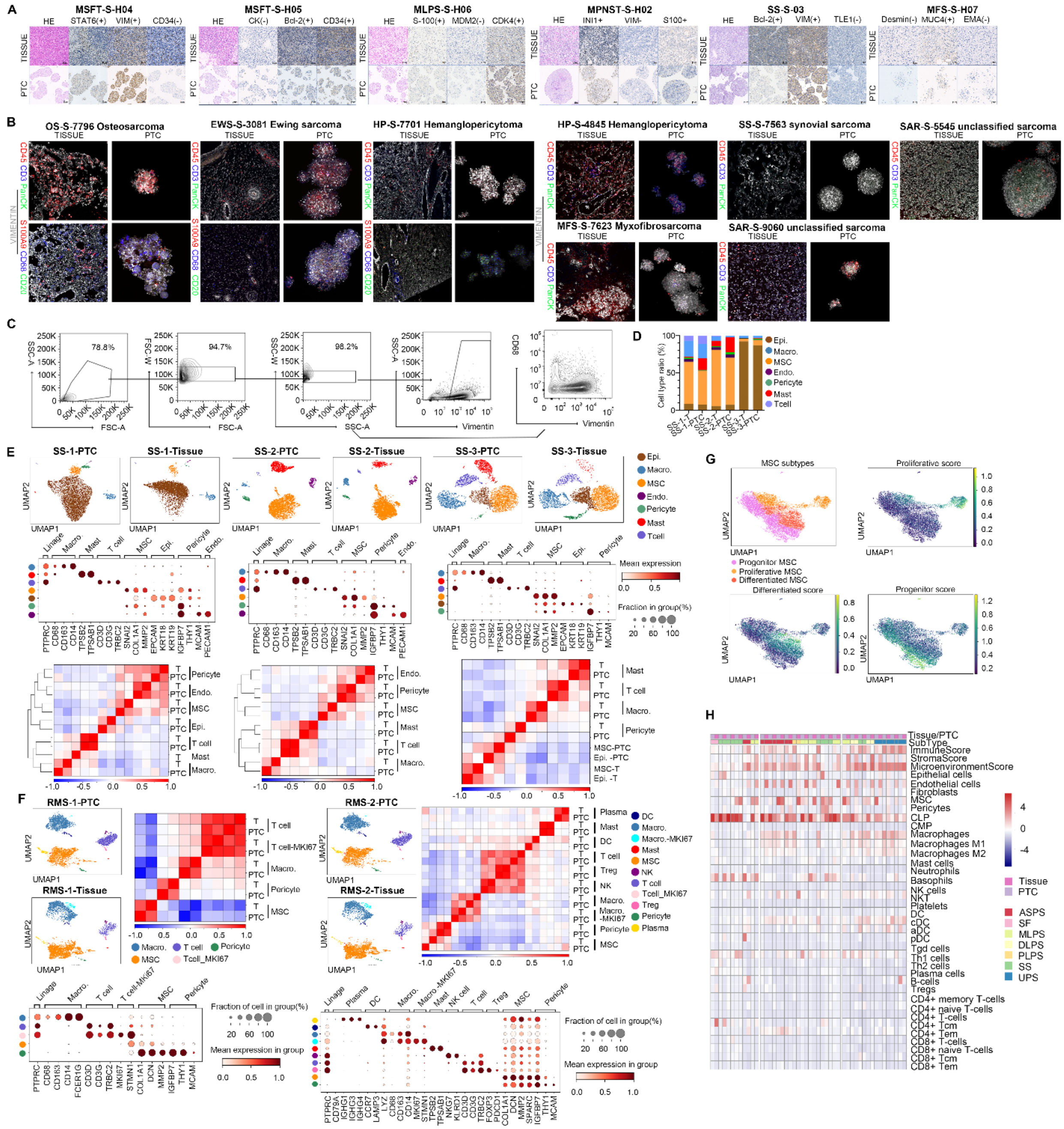

Figure S3. Cellular and Histological Comparison between PTCs and Parental Tumors (related to Figure 3).

(**A**) H&E-stained and Immuno-histochemical (IHC) staining images of tumors and corresponding PTCs deriving from 6 patients with MSFT, MLPS, MPNST, SS, MFS. Scale bars, 50 μm.

(**B**) IF staining for fibroblast marker (VIM$^+$, or vimentin), immune cells marker (CD45$^+$), T cell marker(CD3$^+$), epithelial cell marker (PanCK$^+$), macrophage cell marker (CD68$^+$), B cell marker (CD20$^+$) and neutrophil cell (S100A9$^+$) in parental tumors (left) and corresponding PTCs (right). Scale bars, 100 μm.

(**C**) Gating strategy of flow cytometry used in Figure 3C.

(**D**) Cell type ratio of SS in SS-1, SS-2 and SS-3 parental tumors and PTC clusters. T stands for tissue.

(**E**) UMAP plots of single-cell RNA expression of SS in SS-1, SS-2 and SS-3 parental tumors and PTC clusters.

(**F**) UMAP plots of single-cell RNA expression of RMS in RMS-1 and RMS-2 parental tumors and PTC clusters.

(**G**) UMAP plots of RMS tumor cells in progenitor, proliferating, and differentiated clusters and their score.

(**H**) xCell result of the transcriptomes of STS tumor and PTCs.

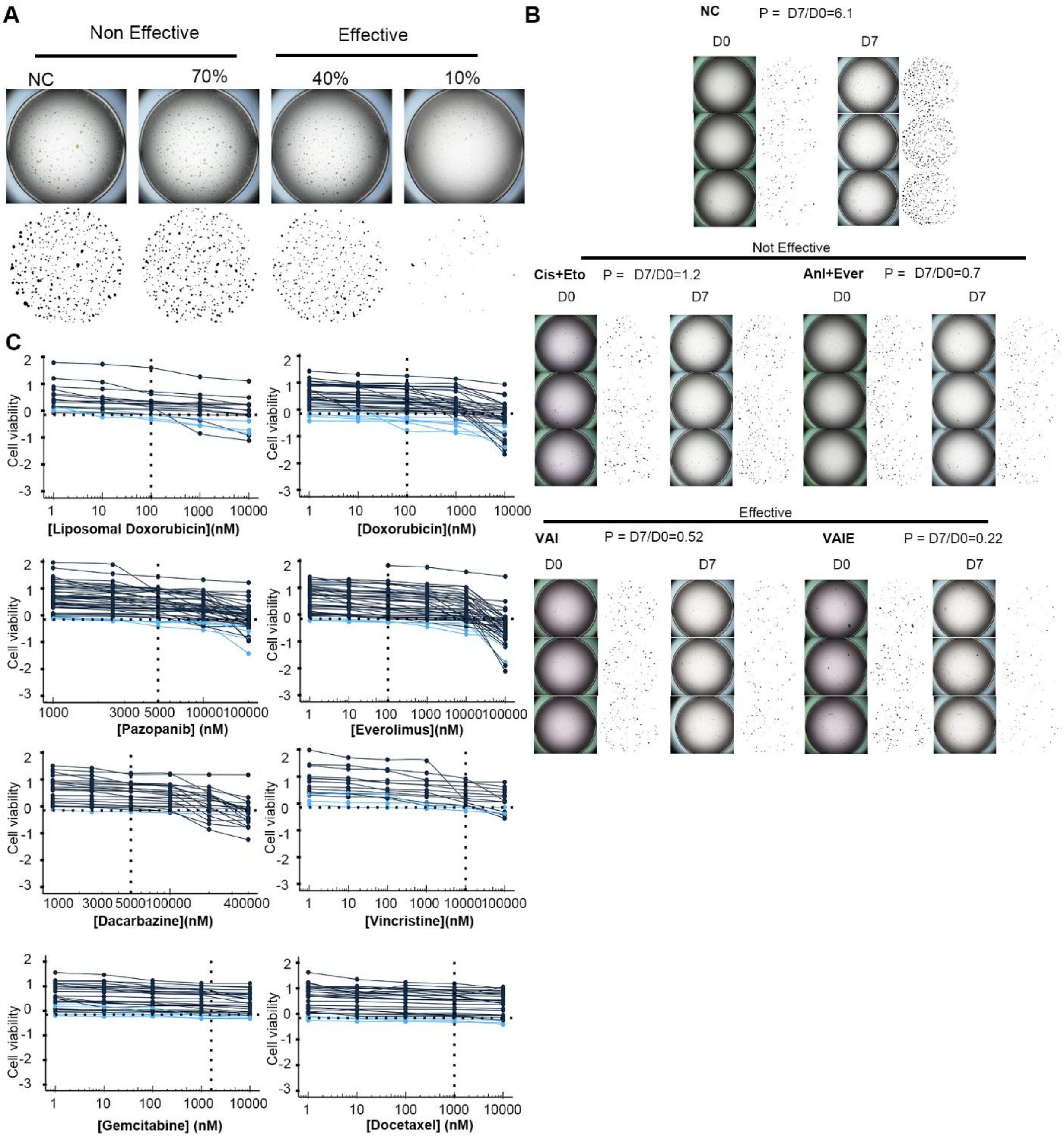

Figure S4. Development of PTC Drug Testing Assay (related to Figure 4).

(**A**) An illustration of cell viability categories. No effect: $\mathbf{p_A} \geq 0.7$; effective: $\mathbf{p_A} < 0.7$. Only clusters with diameters greater than 40 micrometers were selected to calculate the areas. Scale bar: 1 mm.

(**B**) Examples of effective drug and not effective with the area assay applied here. N = 3.

(**C**) Drug Ec for chemotherapy. The determination of Ec was based upon the efficacy rate (ER) closest to the overall response rate (ORR). Efficacy rate referred to the ratio of the number of patients having PTC cell viability < 0.7 to the total patient numbers.

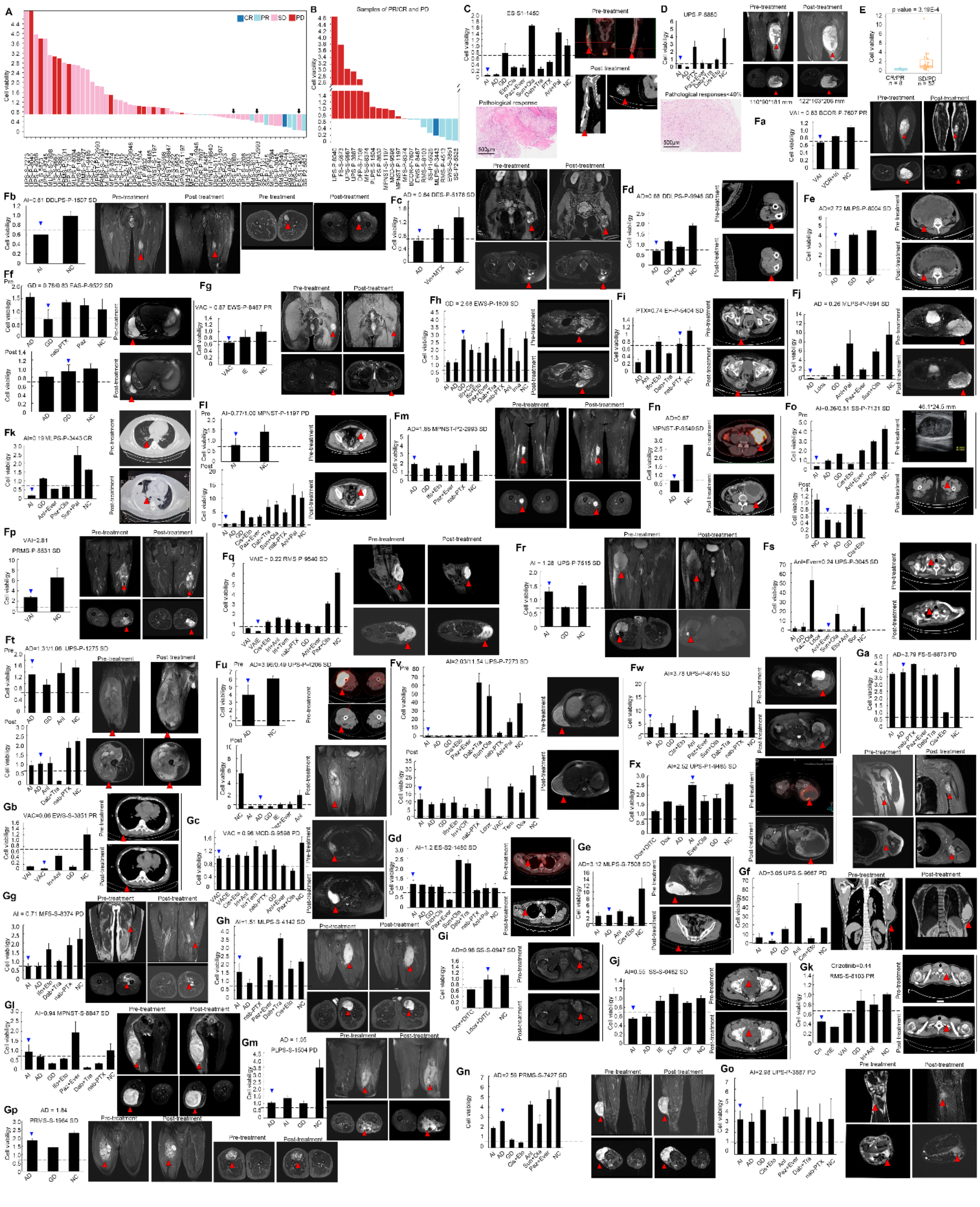

Figure S5. PTCs Recapitulating the Treatment Response of Patients with Sarcoma (related to Figure 5).

(**A**) Waterfall plot illustrating the cell viability based on PTC drug-testing assay and the clinical outcomes of the CR/PR, SD and PD groups. Black arrows indicate pathologic response results of these samples complement clinical imaging and are consistent with PTC.

(**B**) Waterfall plot illustrating the cell viability based on PTC drug-testing assay and the clinical outcomes of the CR/PR and PD groups.

(**C**) The drug-response profile of PTCs from patient UPS-S1-1450 (n=3, data are represented as means ± STD). Blue arrows indicate PTC results of Liposome doxorubicin and Ifosfamide (AI) (left). Selected CT images of patient UPS-S1-1450 before and after the clinical treatment of Liposome doxorubicin and Ifosfamide (AI) regimens (right). Histopathological examination is also shown (left down). Scale bar, 500 µm.

(**D**) The drug-response profile of PTCs from patient UPS-P-5880 (n=3, data are represented as means ± STD). Blue arrows indicate PTC results of Liposome doxorubicin and Ifosfamide (AI) (left). Selected CT images of patient UPS-P-5880 before and after the clinical treatment of Liposome doxorubicin and Ifosfamide(AI) regimens (right). Histopathological examination is also shown (left down). Scale bar, 500 µm.

(**E**) Box-plot of 60 drug test results in the CR/PR group (*n* = 8) and SD/PD group (n = 52) on the basis of PTCs. $P_{CRPR/SDPD} = 3.19\times10^{-4}$.

(**F**) PTCs recapitulate the treatment response of patient in prospective group. Selected CT images of patient before and after treatment were shown beside the drug-response profile (n=3, data are represented as means ± STD). Blue arrows indicate PTC results of the clinical treatment.
(**G**) PTCs recapitulate the treatment response of patient in retrospective group. Selected CT images of patient before and after treatment were shown beside the drug-response profile (n=3, data are represented as means ± STD). Blue arrows indicate PTC results of the clinical treatment.

Table S1 Culture medium and Culture success rate of sarcoma PTC (related to Figure 1).

Component	Concentration
EGF	40 ng/mL
FGF-basic	20 ng/mL
Y-27632-07	10 μM
HGF	30 ng/mL
GlutaMAX	2 nM
Penicillin	100 U/mL
B-27 additive	Diluted to 1X
MEM Non-Essential Amino Acid Solution	Diluted to 1X

Subtype	PTC resource			Failed Sample	Total	Success rate
	Surgery Sample	Punture Sample	Ascites Sample			
Undifferentiated pleomorphic sarcomas	25	14	0	1	40	97.50%
Liposarcomas	19	15	0	2	36	94.44%
Synovial sarcoma	14	7	0	0	21	100.00%
Malignant peripheral nerve sheath tumor	8	7	0	0	15	100.00%
Rhabdomyosarcoma	9	5	1	0	15	100.00%
Myxofibrosarcoma	9	6	0	2	17	88.24%
Alveolar soft part sarcoma	9	2	0	0	11	100.00%
Osteosarcoma	5	1	0	1	7	85.71%
Ewing sarcoma	3	7	0	0	10	100.00%
Epithelioid sarcoma	5	1	0	0	6	100.00%
Others	23	12	0	2	37	94.59%
Unknow	19	15	0	5	39	87.18%
Total	148	92	1	13	254	94.88%

Table S3 Cell components of flow cytometry (related to Figure 3).

	VIM+ cells(VIM+）%	Epithelial (CD326+) %	T cells (CD3+CD45+) %	Macrophage (CD68+)%	B cells (CD20+) %	Dendritic cells (CD11c+) %
Patient-2351_PTC	37.7	0.51	0.095	0.92	0.46	0.12
Patient-2351_T	89.4	0.02	0.28	1.04	0	0.051
Patient-9742_PTC	2.26	0	0.97	1.02	0.01	0.1
Patient-9742_T	1.28	0	0.13	0.4	0	0.021
Patient-8217_PTC	30.3	0	3.46	4.78	0	0.22
Patient-8217_T	77.2	0.01	2.3	1.91	0.01	0.047
Patient-1148_PTC	14	0.019	0	5.71	0.081	1.63

Patient-1148_T	54.5	0.76	0	17.4	0.8	8.78
Patient-4139_PTC	17.8	0.19	0.22	21.1	0.72	6.16
Patient-4139_T	50	0.49	0.015	17.1	0.23	10.5
Patient-9274_PTC	50.3	0.065	0.097	25.8	0	3.22
Patient-9274_T	56.3	0.3	0.077	30.4	0.2	6.55
Patient-6604_PTC	42.2	1.28	0.38			
Patient-6604_T	60.4	0.35	0.011			

Table S4 Efficacy concentration of drugs used in this study (related to Figure 4).

Regimens	Overall response rate (ORR)	Efficacy rate (ER) in PTC	Efficacy concentration [μmol/L]	Reference
Liposomal Doxorubicin	12%-14%	15.8% (3/19)	0.1	[S1-S3]
Doxorubicin	12-15.07%	15% (6/39)	0.1	[S1,S4]
Gemcitabine	18%	17% (5/30)	1.6	[S5,S6]
Docetaxel	10.7%-16%	10% (3/30)	1	[S6,S7]
Anlotinib	10%-13%	16.3% (10/61)	10	[S4,S5]
Everolimus	2%	8.7% (4/46)	0.1	[S8]
Pazopanib	9%	10.4% (5/48)	5	[S9]
Cisplatin	12%-19%	22% (6/27)	5	[S10,S11]
Dacarbazine	4%-7.5%	4.5% (1/22)	50	[S12,S13]
Vincristine	28.6%-31%	20% (3/15)	10	[S14,S15]

Note: the same concentrations were applied to the PTC assay regardless of whether the drug was tested as a singlet or a doublet.

Supplemental references

[S1] Judson, I., Radford, J.A., Harris, M., Blay, J.Y., van Hoesel, Q., le Cesne, A., van Oosterom, A.T., Clemons, M.J., Kamby, C., Hermans, C., et al. (2001). Randomised phase II trial of pegylated liposomal doxorubicin (DOXIL/CAELYX) versus doxorubicin in the treatment of advanced or metastatic soft tissue sarcoma: a study by the EORTC Soft Tissue and Bone Sarcoma Group. Eur J Cancer *37*, 870-877. 10.1016/s0959-8049(01)00050-8.

[S2] Toma, S., Tucci, A., Villani, G., Carteni, G., Spadini, N., and Palumbo, R. (2000). Liposomal doxorubicin (Caelyx) in advanced pretreated soft tissue sarcomas: a phase II study of the Italian Sarcoma Group (ISG). Anticancer Res *20*, 485-491.

[S3] Skubitz, K.M. (2003). Phase II trial of pegylated-liposomal doxorubicin (Doxil) in sarcoma. Cancer Invest *21*, 167-176. 10.1081/cnv-120016412.

[S4] Tian, Z., Yang, Y., Yang, Y., Zhang, F., Li, P., Wang, J., Yang, J., Zhang, P., Yao, W., and Wang, X. (2020). High cumulative doxorubicin dose for advanced soft tissue sarcoma. BMC Cancer *20*, 1139. 10.1186/s12885-020-07663-x.

[S5] Patel, S.R., Gandhi, V., Jenkins, J., Papadopolous, N., Burgess, M.A., Plager, C., Plunkett, W., and Benjamin, R.S. (2001). Phase II clinical investigation of gemcitabine in advanced soft tissue sarcomas and window evaluation of dose rate on gemcitabine triphosphate accumulation. J Clin Oncol *19*, 3483-3489. 10.1200/JCO.2001.19.15.3483.

[S6] Verweij, J., Lee, S.M., Ruka, W., Buesa, J., Coleman, R., van Hoessel, R., Seynaeve, C., di Paola, E.D., van Glabbeke, M., Tonelli, D., and Judson, I.R. (2000). Randomized phase II study of docetaxel versus doxorubicin in first- and second-line chemotherapy for locally advanced or metastatic soft tissue sarcomas in adults: a study of the european organization for research and treatment of cancer soft tissue and bone sarcoma group. J Clin Oncol *18*, 2081-2086. 10.1200/JCO.2000.18.10.2081.

[S7] Zwerdling, T., Krailo, M., Monteleone, P., Byrd, R., Sato, J., Dunaway, R., Seibel, N., Chen, Z., Strain, J., Reaman, G., and Children's Oncology, G. (2006). Phase II investigation of docetaxel in pediatric patients with recurrent solid tumors: a report from the Children's Oncology Group. Cancer *106*, 1821-1828. 10.1002/cncr.21779.

[S8] Yoo, C., Lee, J., Rha, S.Y., Park, K.H., Kim, T.M., Kim, Y.J., Lee, H.J., Lee, K.H., and Ahn, J.H. (2013). Multicenter phase II study of everolimus in patients with metastatic or recurrent bone and soft-tissue sarcomas after failure of anthracycline and ifosfamide. Invest New Drugs *31*, 1602-1608. 10.1007/s10637-013-0028-7.

[S9] van der Graaf, W.T., Blay, J.Y., Chawla, S.P., Kim, D.W., Bui-Nguyen, B., Casali, P.G., Schoffski, P., Aglietta, M., Staddon, A.P., Beppu, Y., et al. (2012). Pazopanib for metastatic soft-tissue sarcoma (PALETTE): a randomised, double-blind, placebo-controlled phase 3 trial. Lancet *379*, 1879-1886. 10.1016/S0140-6736(12)60651-5.

[S10] Sordillo, P.P., Magill, G.B., Brenner, J., Cheng, E.W., Dosik, M., and Yagoda, A. (1987). Cisplatin. A phase II evaluation in previously untreated patients with soft tissue sarcomas. Cancer *59*, 884-886. 10.1002/1097-0142(19870301)59:5<884::aid-cncr2820590504>3.0.co;2-k.

[S11] Brenner, J., Magill, G.B., Sordillo, P.P., Cheng, E.W., and Yagoda, A. (1982). Phase II trial of cisplati(CPDD) in previously treated patients with advanced soft tissue sarcoma. Cancer *50*, 2031-2033. 10.1002/1097-0142(19821115)50:10<2031::aid-cncr2820501010>3.0.co;2-z.

[S12] Garcia-Del-Muro, X., Lopez-Pousa, A., Maurel, J., Martin, J., Martinez-Trufero, J., Casado, A., Gomez-Espana, A., Fra, J., Cruz, J., Poveda, A., et al. (2011). Randomized phase II study comparing gemcitabine plus dacarbazine versus dacarbazine alone in patients with previously treated soft tissue sarcoma: a Spanish Group for Research on Sarcomas study. J Clin Oncol *29*, 2528-2533. 10.1200/JCO.2010.33.6107.

[S13] Zucali, P.A., Bertuzzi, A., Parra, H.J., Campagnoli, E., Quagliuolo, V., and Santoro, A. (2008). The "old

drug" dacarbazine as a second/third line chemotherapy in advanced soft tissue sarcomas. Invest New Drugs *26*, 175-181. 10.1007/s10637-007-9086-z.

[S14] Kellie, S.J., Koopmans, P., Earl, J., Nath, C., Roebuck, D., Uges, D.R., and De Graaf, S.S. (2004). Increasing the dosage of vincristine: a clinical and pharmacokinetic study of continuous-infusion vincristine in children with central nervous system tumors. Cancer *100*, 2637-2643. 10.1002/cncr.20220.

[S15] Defachelles, A.S., Bogart, E., Casanova, M., Merks, J.H.M., Bisogno, G., Calareso, G., Gallego Melcon, S., Gatz, S.A., Le Deley, M.C., McHugh, K., et al. (2021). Randomized Phase II Trial of Vincristine-Irinotecan With or Without Temozolomide, in Children and Adults With Relapsed or Refractory Rhabdomyosarcoma: A European Paediatric Soft Tissue Sarcoma Study Group and Innovative Therapies for Children With Cancer Trial. J Clin Oncol *39*, 2979-2990. 10.1200/JCO.21.00124.
